# Modulating Optoelectronic Properties of TBQP-Based Covalent Organic Frameworks for Chemical Sensing

**DOI:** 10.1021/acsomega.6c02636

**Published:** 2026-06-16

**Authors:** Levy Alvarenga Galindo, Ricardo Paupitz, Augusto Batagin-Neto

**Affiliations:** † School of Sciences, POSMAT, 28108 São Paulo State University (UNESP), Bauru, São Paulo 17033-360, Brazil; ‡ Institute of Geosciences and Exact Sciences, São Paulo State University (UNESP), Rio Claro, São Paulo 13506-900, Brazil; § Institute of Sciences and Engineering, São Paulo State University (UNESP), Itapeva, São Paulo 18409-010, Brazil

## Abstract

Covalent organic frameworks (COFs) have emerged as promising porous organic semiconductors for chemical sensing, owing to their structurally tunable electronic properties and accessible pores. A key challenge for the rational design of these materials is establishing a clear understanding of how molecular-level functionalization dictates their optoelectronic response. Here, we address this challenge through a computational investigation of TBQP-based COFs, combining density functional theory and molecular dynamics to investigate the impact of edge functionalization on their electronic structure, optical properties, and adsorption reactivity toward Cl_2_, ClF_3_, and SO_2_. Using representative molecular models and fragments derived from the TBQP architecture, our results demonstrate that the Frontier orbital energies and band gap of the extended framework are primarily governed by the electronic character of the constitutive building blocks. This enables systematic property modulation through targeted substituent effects. We establish quantitative correlations between experimental Hammett parameters, DFT-based reactivity indices, and analyte-induced electronic perturbations, providing descriptors that link chemical modification to optoelectronic behavior. Furthermore, we find that analyte adsorption can introduce midgap states, revealing a general electronic sensing mechanism for π-conjugated COFs. These findings not only offer practical guidelines for tailoring the electronic and reactive properties of TBQP frameworks but also underscore the critical role of molecular functionalization in governing the sensing performance of porous organic semiconductors. The identified structure–property relationships provide useful guidelines for the rational design of COF-based sensors.

## Graphical abstract

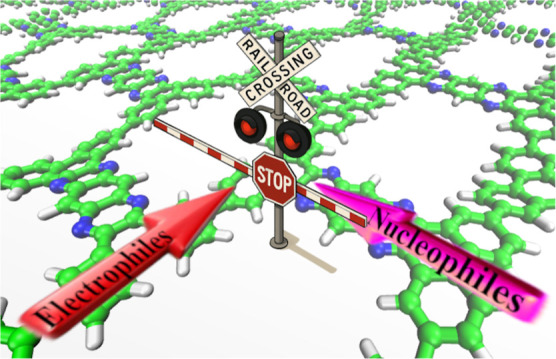

## 1 Introduction

Covalent organic frameworks (COFs) have emerged as an important class of crystalline porous materials with tunable electronic and structural properties.^1^ These materials can be synthesized as two-dimensional (2D) or three-dimensional (3D) architectures, typically exhibiting high specific surface areas, low skeletal densities, and remarkable thermal and chemical stabilities.^2^ Owing to their intrinsic porosity and readily accessible active sites, COFs have attracted considerable attention for applications in energy storage^3^, catalysis and redox processes,^4^ and gas separation.^4,5^

Due to their inherent structural tunability, the rational design of COFs has evolved far beyond mere geometric optimization of pore sizes and surface areas. In recent years, a rapidly growing body of literature has demonstrated the potential of systematically engineering the electronic structure of COFs to enable specific, high-performance functionalities. Such precise electronic modulations have proven critical in advancing COFs as efficient heterogeneous catalysts, particularly for demanding processes such as photocatalytic water splitting, oxygen evolution reactions, and electrocatalytic reductions.^6–9^ Furthermore, the strategic alignment of their Frontier molecular orbitals via targeted functionalization continues to drive recent advances in optoelectronics and selective chemical sensing.^10–12^

Despite these significant advances, establishing systematic and quantitative relationships between molecular-level functionalization and the resulting electronic and reactive properties of COFs remains a challenge.^13^ In particular, predictive frameworks that directly connect substituent effects to optoelectronic response and analyte interactions are still relatively limited.

Building upon these established principles, in this study we investigate a rectangular COF constructed from 2,7,13,18-tetrabromodibenzo­[*a*,*c*]­dibenzo­[5,6:7, 8]­quinoxalino­[2,3-*i*]­phenazine units (TBQP-COF), previously reported by Liu et al., a promising organic anode material for sodium-ion batteries.^3^ Using density functional theory (DFT)-based calculations and fully atomistic reactive molecular dynamics (FARMD) simulations, we modeled and analyzed representative molecular building blocks and reduced two-dimensional repeating subunits of the TBQP-COF structure. Our objective is to establish systematic strategies for tuning the electronic energy levels of these materials by evaluating how distinct functional side groups influence their optoelectronic properties, local reactivity, adsorption energetics, and dynamic interactions with gaseous analytes relevant to chemical sensing. In particular, the combined DFT/FARMD approach enables the correlation of localized electronic descriptors with the preferential adsorption behavior and the spatial distribution of analytes around the COF pores. The results provide atomistic-level insights into how edge functionalization modulates the electronic structure and host–guest interactions in TBQP-based COFs, thereby offering useful design principles for the development of selective and tunable COF-based chemical sensors.

## 2 Material

Figure 1 shows the basic building structures of TBQP-COF from isolated and fused rings to the edge/corner unit combinations and the simple basic unit (1D-CAP) of TBQP-COF. More extended structures are presented in Figure 2, which were designed according to subunits proposed by Liu et al.^3^ (keeping the same nomenclature for simplicity): (a) 1D-CAP, (b) 2D-CAP, and (c) 2D-CAP-2. Distinct side groups were attached to the 2D-CAP (R group), selected based on their Hammett constants (σ).^14^ These values quantify the electron-donating/withdrawing character of the groups via inductive and resonance effects, enabling systematic modulation of the compound’s electronic structure (Table 1).

**Figure 1:**
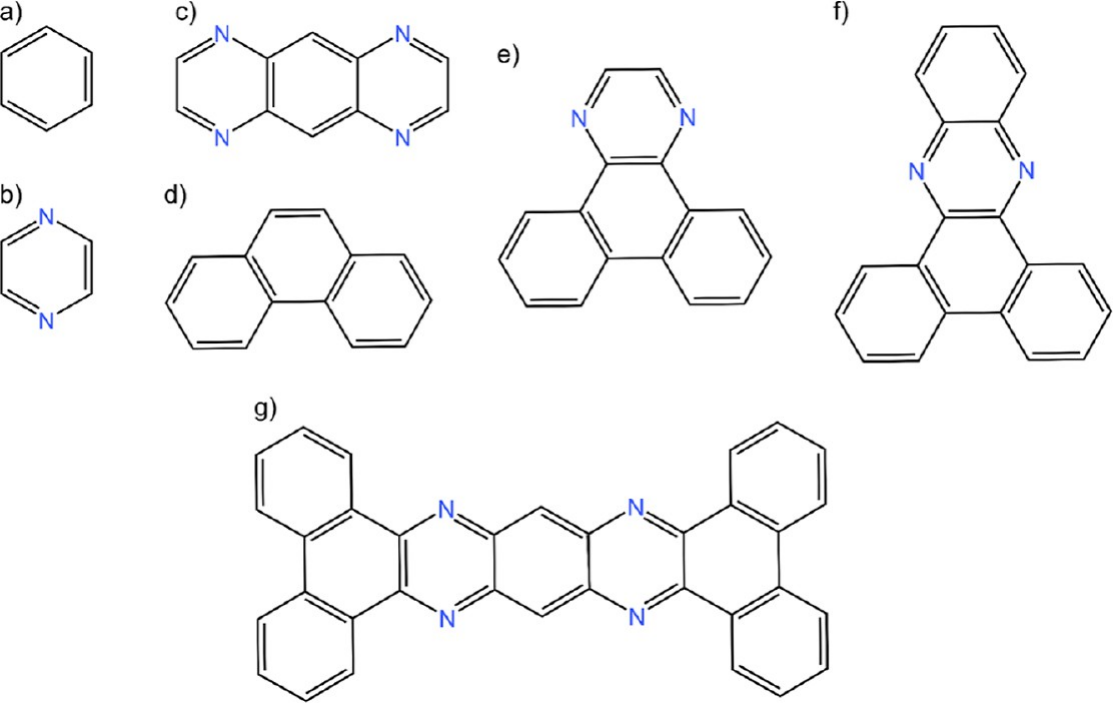
Basic structures of TBQP-COF: (a) ring 1, (b) ring 2, (c) edge unit, (d) corner unit, (e) corner unit + ring 2, (f) corner unit + ring 2 + ring 2, and (g) basic repeating unit of TBQP-COF (1D-CAP).

**Figure 2:**
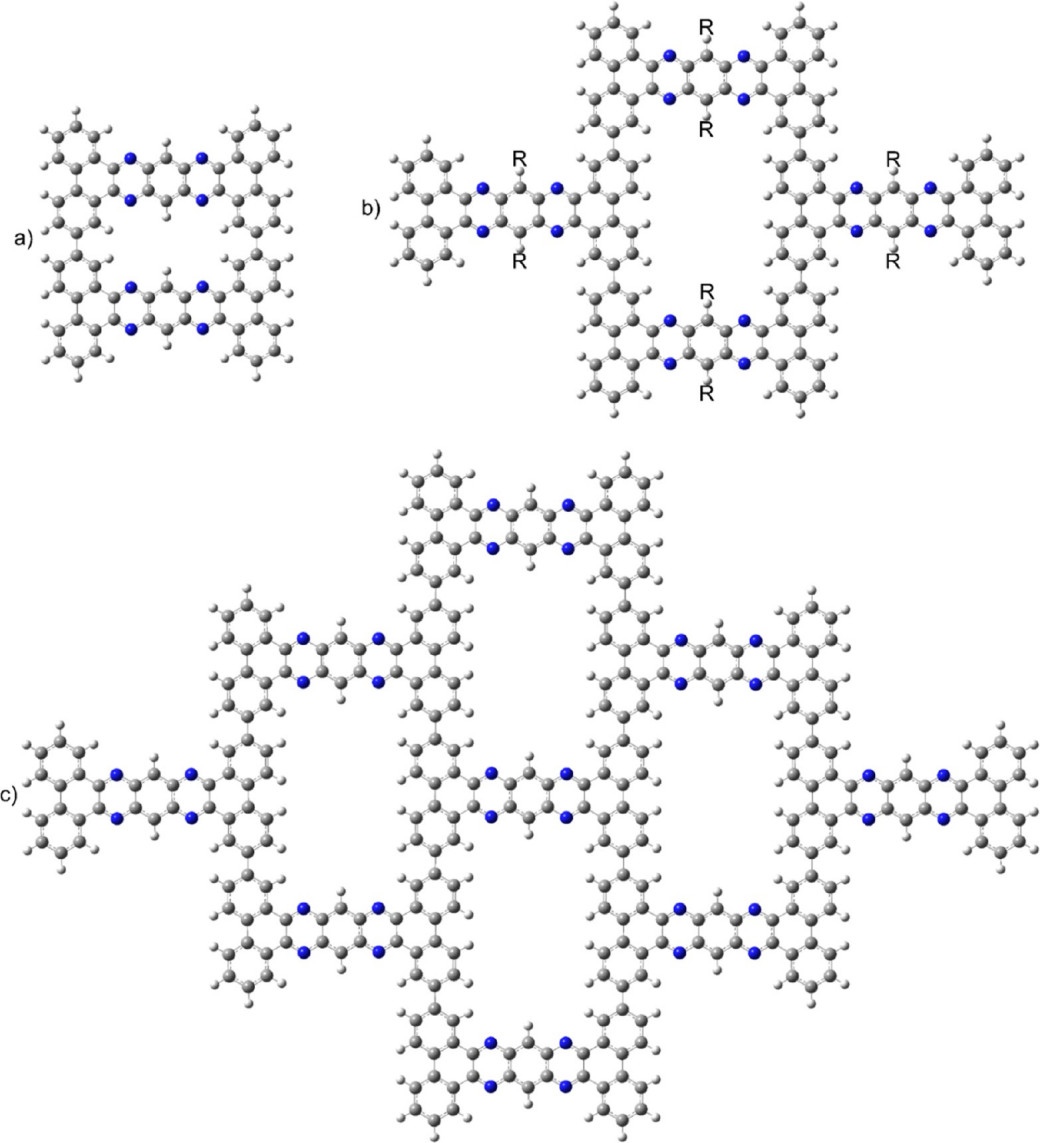
Basic structures of TBQP-COFs: (a) 1D-CAP (2 units), (b) 2D-CAP, and (c) 2D-CAP-2.

**Table 1:** Side Groups and Hammett Indices: Resonance (σ_R_) and Inductive (σ_I_) Effects

	side group (R)									
Hammett indices	–H	–C≡CH	–CH_3_	–C_6_H_5_	–C≡N	–F	–NH_2_	–NO_2_	–OCH_3_	–OH
σ_I_	0.00	0.29	–0.01	0.12	0.57	0.54	0.17	0.67	0.30	0.24
σ_R_	0.00	–0.04	–0.16	–0.11	0.08	–0.48	–0.80	0.10	–0.58	–0.62

## 3 Methodology

All the structures were designed with the aid of the Gaussview computational package.^15^ Initial geometry optimizations were performed at the Hartree–Fock (HF) level with the PM3 semiempirical Hamiltonian,^16^ followed by full ground-state optimizations via density functional theory (DFT) using the Gaussian 16 package.^17^ DFT-based calculations were conducted with the B3LYP exchange–correlation functional^18–21^ and 6-31G­(d,p) polarized basis set on all the atoms. This level of theory was selected for its proven accuracy in describing the electronic properties of organic frameworks^22^ and its favorable cost–benefit ratio.^23^ A fragment-based TD-DFT approach was adopted to investigate localized host–guest interactions and analyte-induced electronic perturbations in TBQP-COFs. Although this methodology does not explicitly account for interlayer π–π stacking or band-dispersion effects present in periodic COF structures, it provides a reliable description of localized excitations and charge-transfer processes governing the sensing response and has been successfully employed in studies of COF-based systems.^24–28^ Therefore, while some absolute quantitative properties may differ from those of the bulk material, the relative trends associated with substituent effects and analyte interactions are expected to remain qualitatively consistent.

Local chemical reactivities were evaluated via condensed-to-atoms Fukui indices (CAFIs),^29,30^ using finite differences of electronic populations, as reported elsewhere.^31,32^ These indices identify molecular sites (*k*) that are prone to interact with nucleophiles (*f*_*k*_^+^), electrophiles (*f*_*k*_^–^), and/or free radicals (*f*_*k*_^0^). In this study, CAFIs were evaluated using the Hirshfeld atomic charges^33–35^ to ensure a physically sound description of the electron density distribution, using the same DFT approach of geometry optimization.

Adsorption studies were conducted for selected analytes (Cl_2_, ClF_3,_ and SO_2_) over reactive sites identified in the CAFI analysis. The geometry of adsorbed systems was optimized in a DFT/B3LYP/6-31G­(d,p) approach using the D3 version of Grimme’s dispersion correction.^36^ Environmental effects were simulated using the polarizable continuum model (PCM) with dibutyl ether (ε = 3.0473), reproducing the low-polarity dielectric medium typical of organic frameworks.^37–39^ TD-DFT calculations were carried out in a B3LYP/6-31G­(d,p)/GD3/PCM approach to evaluate the optical responses (considering five singlet transitions). The adsorption energies were estimated with the aid of the Orca 6.0 computational package,^40^ considering the solvent and the counterpoise method to correct the basis set superposition error,^41,42^ as reported elsewhere.^43^

To bridge the gap between localized electronic effects and the dynamic behavior of the bulk material, fully atomistic reactive molecular dynamics (FARMD) simulations were also conducted to evaluate the COF’s adsorption capabilities of selected analytes. A simulation box with 25 × 17 × 50 Å^3^ was defined, considering appropriate periodic boundary conditions (PBCs) in all directions. Promising 2D-CAP-based derivatives (identified from DFT) were placed in the presence of Cl_2_ and SO_2_. Each system was let to evolve using the velocity Verlet algorithm^44,45^ as implemented in LAMMPS software in an NVT ensemble controlling temperature (300 K) by the application of a Nose–Hoover thermostat^46^ for 0.1 ns (with timesteps of 0.1 fs). During the simulations, both the COFs and analyte molecules were allowed to evolve. FARMD calculations were made considering the ReaxFF force field (ZIF-optimized parameters)^47^ with the aid of the LAMMPS computational package.^48^ Radial distribution functions, *g*(*r*), were analyzed with visual molecular dynamics (VMD) software.^49^ Effective interaction color maps were also constructed by evaluating the average intermolecular distances via custom routines developed in Fortran 90 and VMD.

## 4 Results and Discussion

### 4.1 Local Reactivity and Electronic Structure of the TBQP-COF Building Blocks

Figure 3 illustrates the CAFI results obtained for 1D-CAP (2 units), 2D-CAP (1 unit), and 2D-CAP-2 (1 unit) structures (see the Supporting Information for extended systems). Red and blue regions define sites with high and low reactivities, respectively. The other colors indicate regions with intermediate reactivity following an RGB scale. Each structure has its own color scale, thus representing only the intramolecular reactivity.

**Figure 3:**
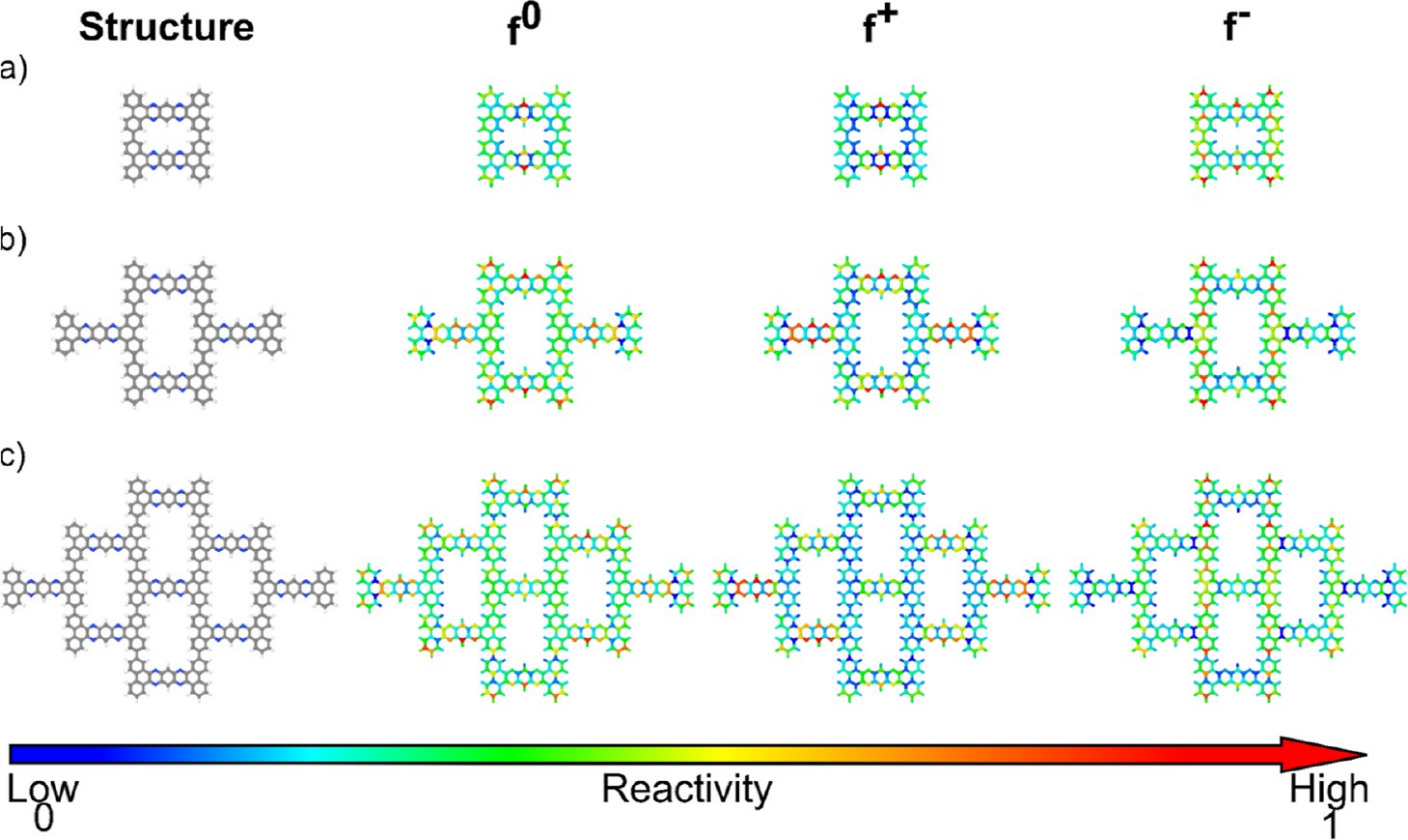
Color maps associated with CAFIs for (a) 1D-CAP, (b) 2D-CAP, and (c) 2D-CAP-2 systems. Atoms in gray, blue, and white define carbon, nitrogen, and hydrogen, respectively. Red regions indicate more reactive sites with respect to free radicals (*f* ^0^), nucleophiles (*f* ^+^), and electrophiles (*f* ^–^).

The results show three main features that are common to all the systems: (i) the reactivity related to *f* ^+^ (in relation to nucleophilic external agents) is located on the horizontal segments of the COFs (edges: azinic structures); (ii) the reactivity related to *f* ^–^ (related to electrophiles) is located on the vertical segments (corner units: biphenyl structures); and (iii) the reactivity of the system is dominated by *f* ^+^, given the greater similarity of this descriptor with *f* ^0^ (the average between *f* ^+^ and *f* ^–^). Note that the trends observed for smaller structures are maintained in the more extended ones, suggesting that reactivity data for these systems can be estimated on the basis of smaller structures associated with 1D-CAP and 2D-CAP.

Given these results, it can also be assumed that the TBQP-COF pores interact more effectively with nucleophilic agents (*f* ^+^) due to the dominance of these indices in the central regions of the pores.

Figure 4 illustrates the spatial distribution of the Kohn–Sham (KS) Frontier molecular orbitals (FMOs, i.e., HOMO and LUMO) over the reduced structures of the TBQP-COFs. Note that the HOMO extends mostly over the vertical segments of the structures, whereas the LUMO extends mostly over the horizontal segments of the structure. Although the LUMO is located over only one horizontal segment (edge) of the more extended structures, it is noticed that other horizontal segments are associated with orbitals around the LUMO (e.g., LUMO+1, LUMO+2, etc.; see Figure S4). Considering that chemical species that tend to gain electrons are prone to interact with the HOMO, while species that tend to lose electrons are supposed to interact with the LUMO, these data reinforce the results obtained by CAFIs.

**Figure 4:**
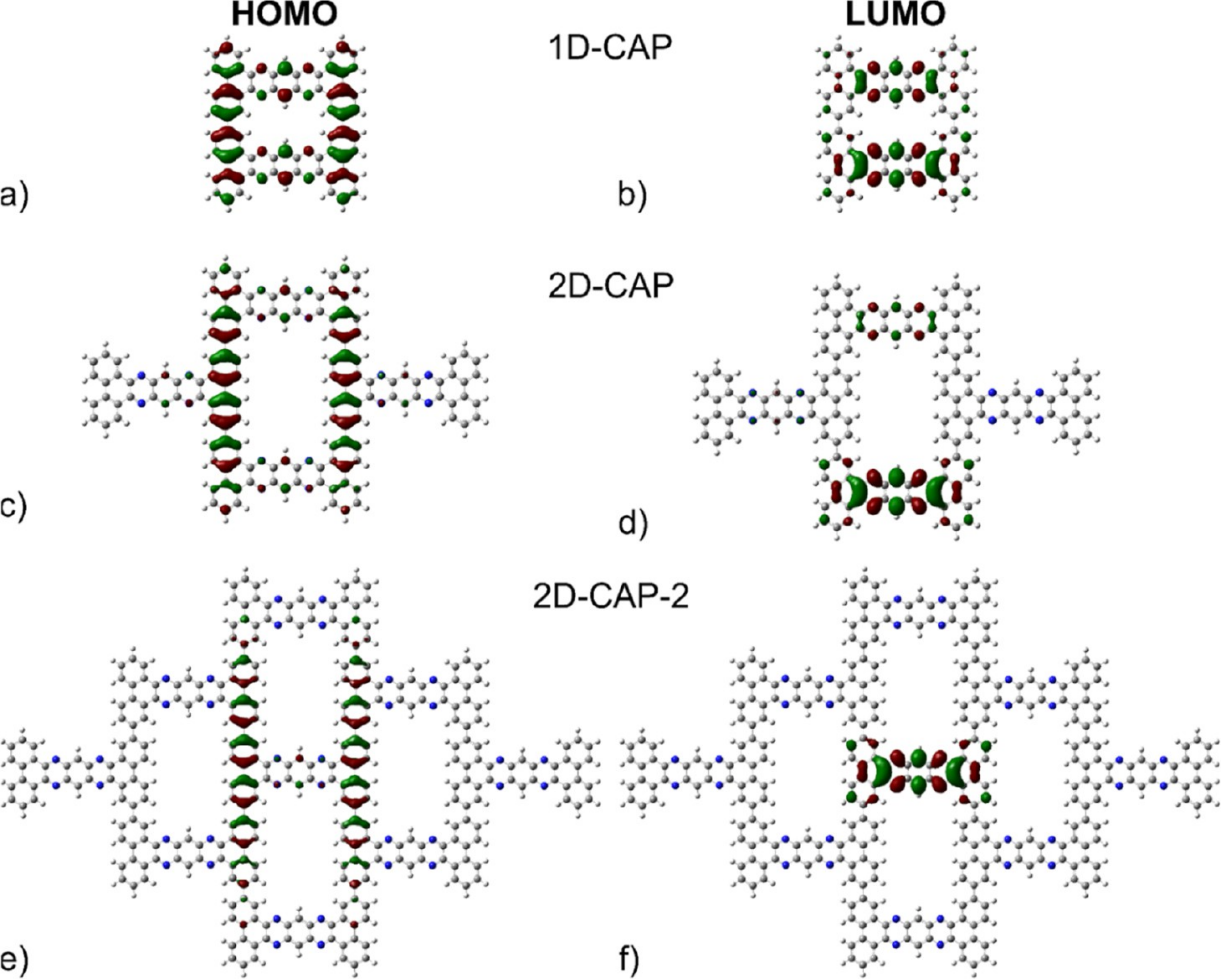
Spatial distribution of the KS-FMOs: (a) HOMO and (b) LUMO of 1D-CAP (2 units); (c) HOMO and (d) LUMO of 2D-CAP; and (e) HOMO and (f) LUMO of 2D-CAP-2.

Figure 5 presents the KS-FMO energies of TBQP-COF building blocks. This analysis was carried out to understand how the FMOs of the COF are generated from the fusion of the distinct basic units. In general, the building blocks were divided into the following structures: ring 1 (unbound basic structure present in the corner and edge units); ring 2 (ring that makes up the edge unit); edge unit; corner unit (vertex); DBzq (COF corner unit, from ring 1 and ring 2 merging), and BzPh (COF corner unit, extended DBzq).

**Figure 5:**
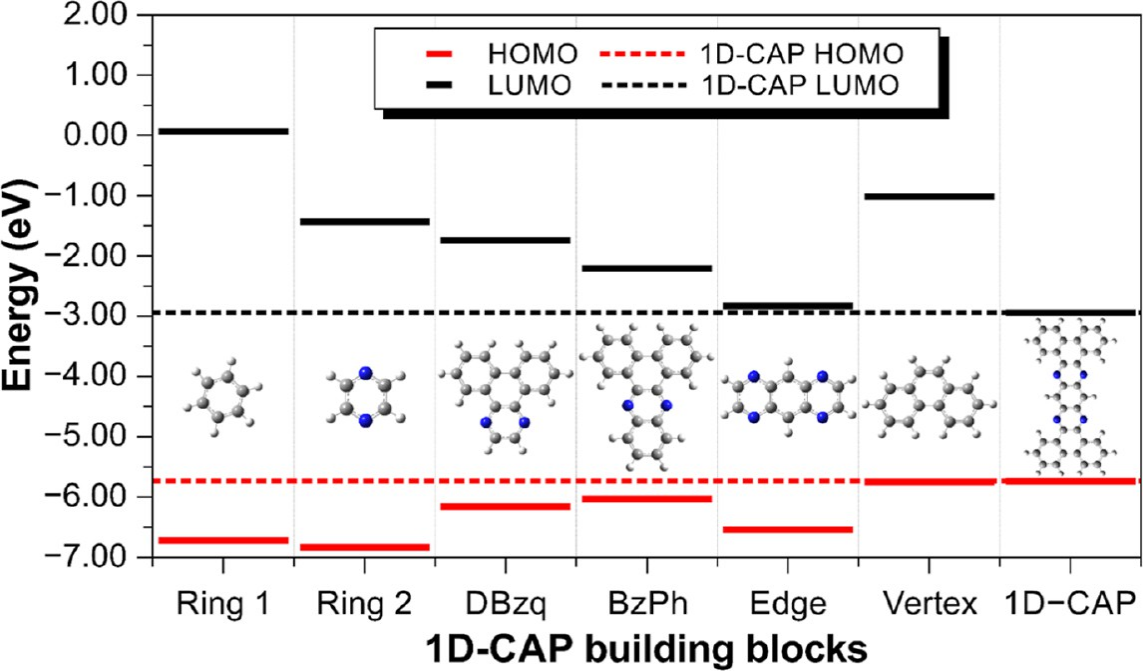
HOMO and LUMO energies of the TBQP-COF 1D-CAP’s building blocks.

A wider gap is noticed for ring 1 (6.8 eV), while the 1D-CAP structure presents the smallest value (2.8 eV). The results also evidence a clear distinction between HOMO and LUMO compositions into the TBQP-COF structure; HOMO being associated with the vertex, while the LUMO is linked to the edge, reinforcing that the electronic structure of this COF can be understood as the superposition of the electronic properties of its horizontal (edge) and vertical (corner) subsegments.

CAFI studies were also conducted for 1D-CAP and its building blocks, aiming to better evaluate the local reactivity of this system. Figure 6 shows the obtained results. It follows the same definition (color scheme and criteria) used in Figure 3.

**Figure 6:**
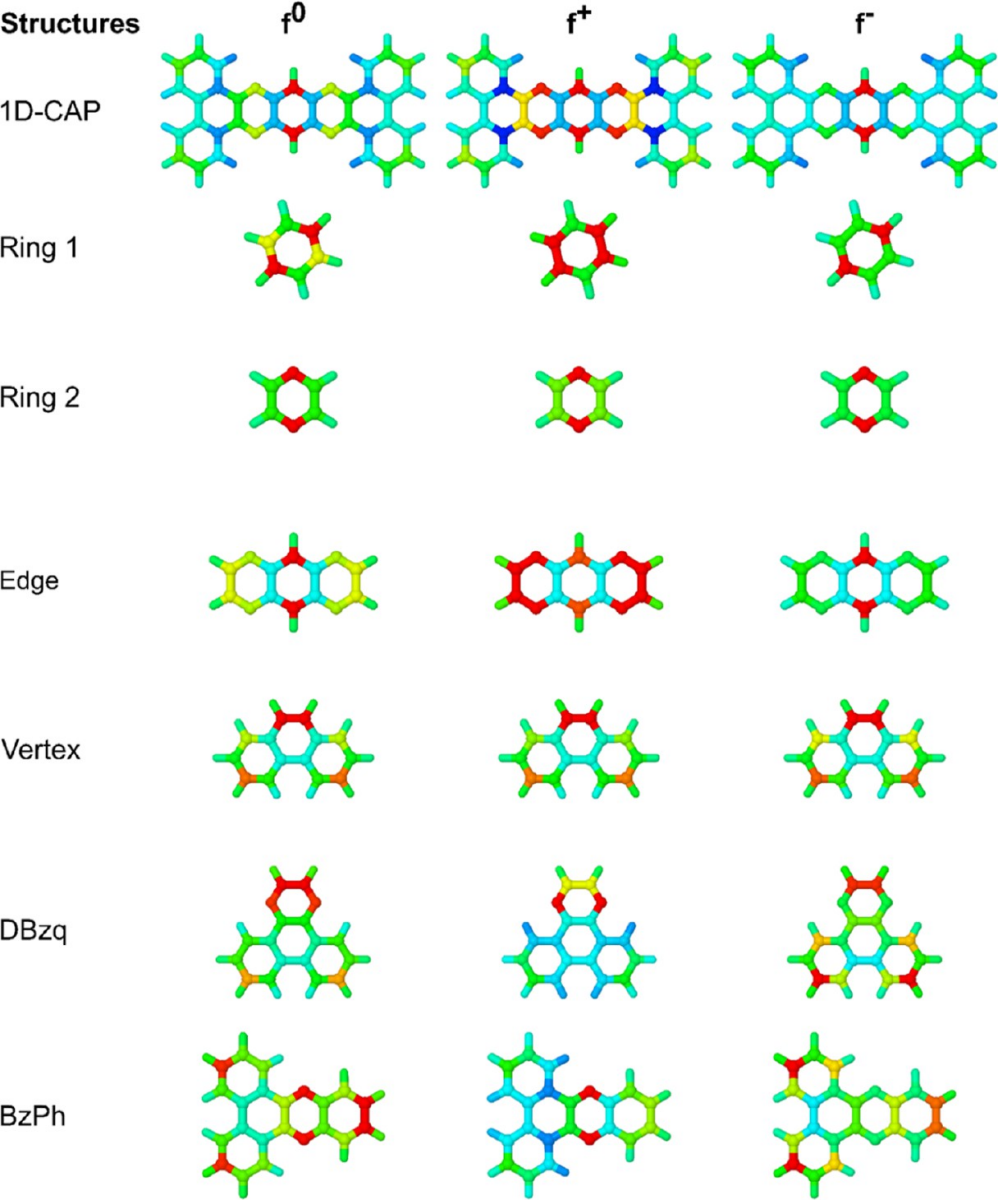
CAFI color maps for TBQP-COF 1D-CAP and its building blocks.

Ring 1 (Bz) presents a reflection symmetry in relation to diametrically opposed atoms, with four (two) main reactive sites for *f* ^+^ (*f* ^–^). Ring 2 (Pz) presents similar reactivities in relation to free radicals, nucleophiles, and electrophiles, which are centered on the nitrogen atoms. The edge unit shows high reactivity toward nucleophiles on terminal carbon atoms and central nitrogens and high reactivity toward electrophiles on the central carbon atoms. The vertex shows similar reactivities toward free radicals, nucleophiles, and electrophiles, especially on carbon atoms. The intermediary structures DBzq and BzPh show high reactivity toward electrophiles on terminal atoms with high reactivity toward nucleophiles on the nitrogen atoms. Finally, 1D-CAP shows high reactivity on the central carbon and nitrogen atoms. In general, the reactivity of the 1D-CAP unit is determined by a combination of the reactivity of its blocks, with the edges dominating the corners. This is different from the data obtained for the more extended structures, which show the dominance of the corners in *f* ^–^ (see Figure 3).

### 4.2 Influence of Ligands on the Electronic Structure of TBQP-COFs

To investigate the influence of ligands on the electronic structure and local reactivity of TBQP-COF, side groups were inserted on the edges of 2D-CAP and 2D-CAP-2. 1D-CAP (2 units) was not considered, given its reduced pore size. Table 2 and Figure 7 illustrate the FMO energies (*E*_HOMO_ and *E*_LUMO_) estimated for the systems.

**Table 2:** HOMO and LUMO Energy Values of the TBQP-COF Structures: 2D-CAP and 2D-CAP-2

systems	substituents	*E*_HOMO_ (eV)	*E*_LUMO_ (eV)	*E*_gap_ (eV)
2D-CAP	H	–5.47	–3.03	2.44
	C_6_H_5_	–5.33	–3.30	2.03
	CCH	–5.42	–3.25	2.17
	CH_3_	–5.36	–2.95	2.41
	CN	–5.76	–3.79	1.97
	F	–5.52	–3.16	2.36
	NH_2_	–4.24	–2.68	1.57
	NO_2_	–5.73	–4.12	1.61
	OCH_3_	–4.78	–2.88	1.89
	OH	–5.02	–3.09	1.93
2D-CAP-2	H	–5.40	–3.12	2.28
	C_6_H_5_	–5.38	–3.40	1.99
	CH_3_	–5.32	–3.04	2.29
	CCH	–5.41	–3.35	2.06
	CN	–5.69	–3.86	1.83
	F	–5.46	–3.26	2.20
	NH_2_	–5.16	–3.09	2.07
	NO_2_	–5.66	–4.18	1.48
	OCH_3_	–4.82	–2.94	1.88
	OH	–5.00	–3.23	1.77

**Figure 7:**
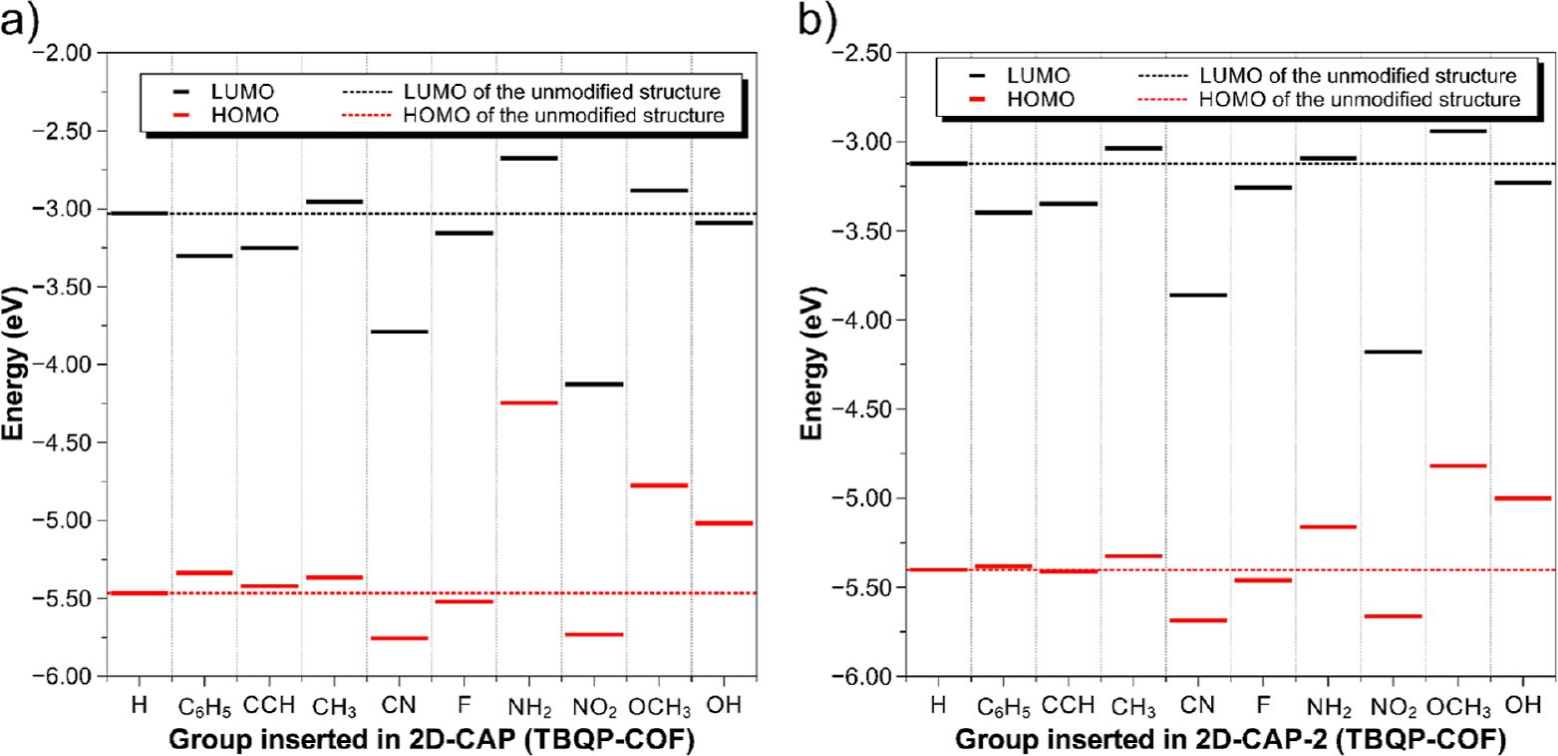
Change in FMO energies for TBQP-COF derivatives with side groups: (a) 2D-CAP and (b) 2D-CAP-2.

Small changes are noticed for C_6_H_5_, CH_3,_ and F, while reduced *E*_gap_ is induced by CN, NH_2_, NO_2_, OCH_3,_ and OH. In particular, the presence of CN, NO_2,_ and F reduces the *E*_HOMO_ values, increasing the system’s chemical stability, while an opposite slight effect is noticed for CH_3_, C_6_H_5_, OH, OCH_3_, and NH_2_ leading to progressive higher *E*_HOMO_ values. The significant effects noticed for 2D-CAP/CN, 2D-CAP/NO_2,_ and 2D-CAP/NH_2_ indicate a high capability of electronic property modulation of TBQP and selectivity tuning with distinct analytes. CN and NO_2_ present significant changes on *E*_HOMO_ and *E*_LUMO_ for both the systems (with a reduced intensity for 2D-CAP-2/NH_2_).

Figure 8 illustrates simplified structure–property rules involving the Hammett indices of the substituents and FMO energies of the TBQP-COF derivatives for the 2D-CAP (a) and 2D-CAP-2 (b) systems.

**Figure 8:**
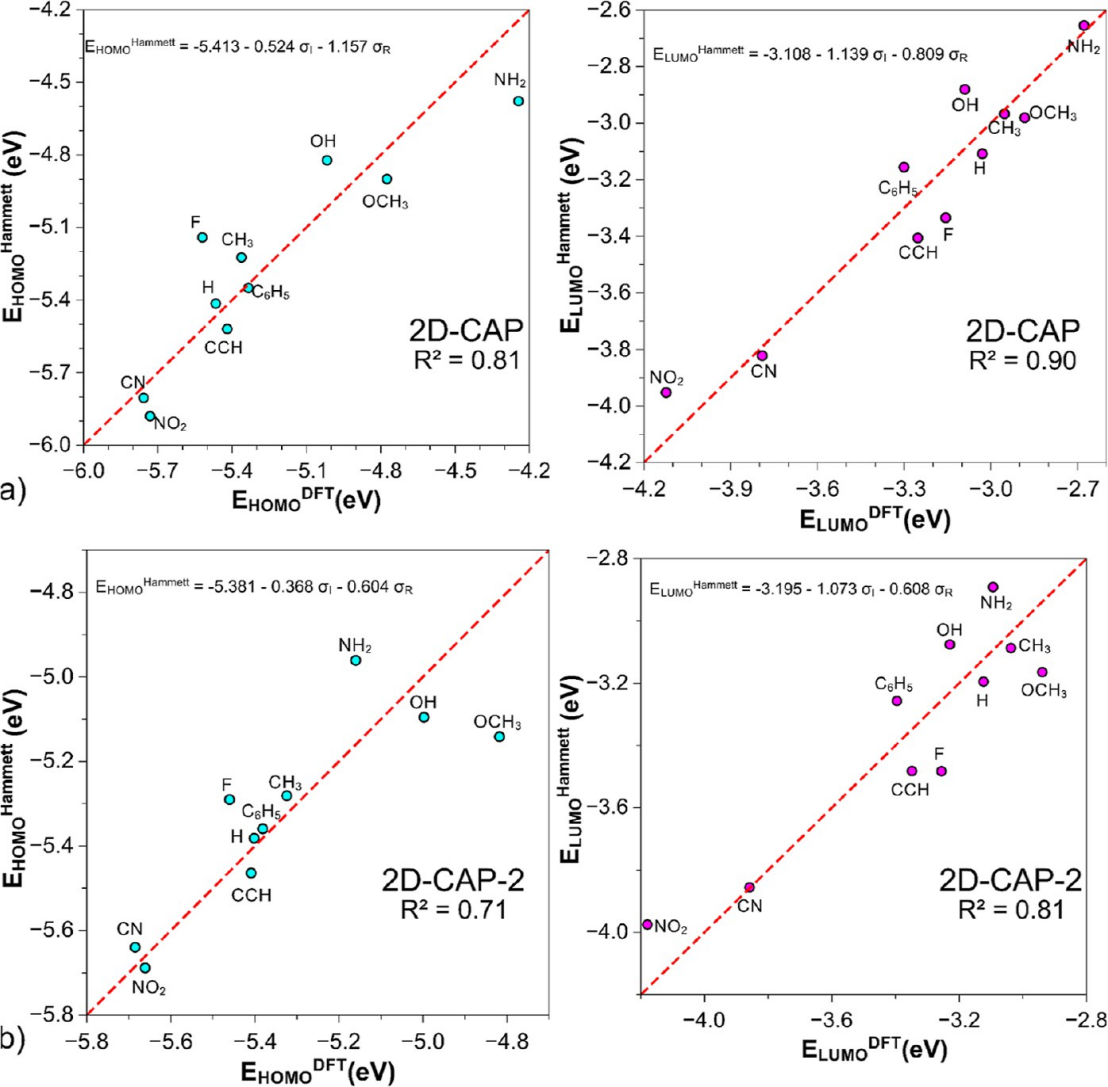
Prediction of the energy of the FMOs as a function of the Hammett indices of the substituents using multiple linear regression: (a) 2D-CAP and (b) 2D-CAP-2.

Multiple linear regression (MLR) equations with reasonable Pearson regression parameters (*R* ^2^) can be underlined for both structures, especially for 2D-CAP. In both cases, the dominance of the σ_R_ parameter over σ_I_ can be seen for the HOMO, as evidenced by its higher coefficient in the MLR. The opposite effect is observed for LUMO (dominance of σ_I_). In general, the obtained equations allow the prediction of electronic features of TBQP-based COFs without expensive DFT calculations and can be used as a first approach for band gap engineering, given the availability of side-group Hammett indices.

Aiming to evaluate the local reactivity of the proposed derivatives, Figure 9 shows the color maps for CAFIs estimated for the different TBQP-based COFs, considering 2D-CAP/R derivatives (see Supporting Information for 2D-CAP-2/R). Again, we follow the same definition used in Figure 3.

**Figure 9:**
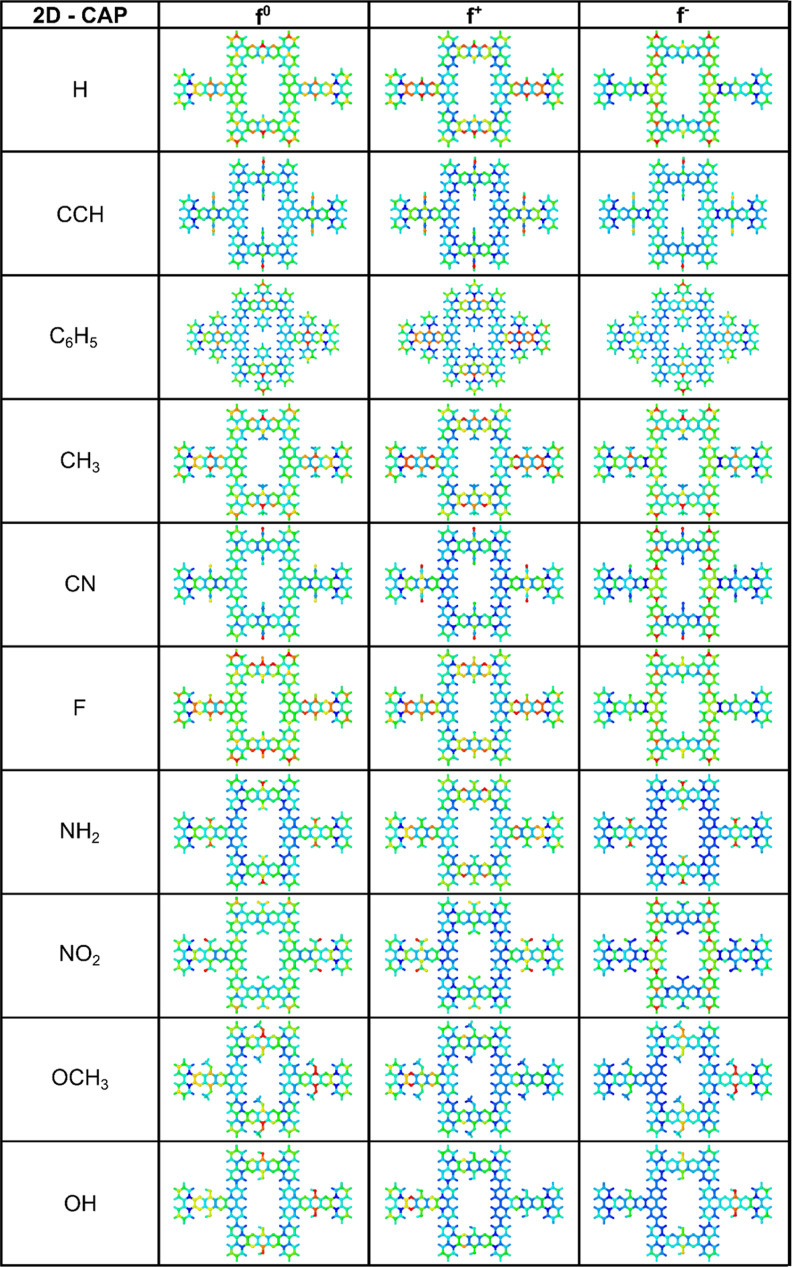
CAFI for TBQP-COF 2D-CAP/R derivatives. R = H, CCH, C_6_H_5_, CH_3_, CN, F, NH_2_, NO_2_, OCH_3,_ and OH.

After replacing the hydrogen of 2D-CAP/H with the different side groups, the following changes can be seen:

- In relation to *f* ^+^: (i) minor changes are observed for R = C_6_H_5_, CH_3_, and F, with the high reactivity maintained on the nitrogen atoms and on the central carbon of the edges (where the side groups are attached); (ii) intensification of the reactivity on the nitrogen atoms of the edges with a drop in the reactivity of the central carbon for R = NH_2_ (symmetrical effect), OCH_3_, OH (asymmetrical effect); and (iii) centralization of the reactivity on the substituents for R = CCH (outermost carbon), CN (nitrogen), and NO_2_ (oxygen).
- In relation to *f* ^–^: (i) minor changes for R = CH_3_ (with intensified reactivity of the central carbon of the edge), F, and NO_2_, where reactivity is still centered on the corner units (vertical structures); (ii) high reactivity on the corner units and on the substituents for R = CN; (iii) centralization of the reactivity on the substituents for R = CCH (outermost carbon), C_6_H_5_ (carbon in the para position), NH_2_ (nitrogen), OCH_3,_ and OH (oxygen).
- In relation to *f* ^0^: (i) minor changes for R = CH_3_ and F; (ii) centralization of reactivity on the substituents for R = CCH (outermost carbon), C_6_H_5_ (carbon in the para position), CN, (nitrogen), NH_2_ (nitrogen), NO_2_, OCH_3_, and OH (oxygen).

Similar effects are noticed for the 2D-CAP-2/R derivatives with more pronounced asymmetries. An exception is the centralization of the *f* ^–^ reactivity on the central edge carbon for R = CH_3_, which is not observed in the 2D-CAP system. Overall, both structures show a predominance of reactivity along the edges compared to the corners (see the Supporting Information for details).

According to the effects induced on the COF, four distinct groups of substituents can be outlined: (i) electron donors with minor effects on the reactivity: R = CH_3_ and CCH; (ii) electron acceptors with a moderate effect on reactivity (slightly deactivating): R = C_6_H_5_, NH_2_, OH, OCH_3_, and CN; (iii) electron acceptors with a significant effect on reactivity (moderately deactivating): R = F; and (iv) electron acceptors with a significant effect on reactivity (strongly activating).

Figure 10 illustrates the spatial distribution of the FMOs (HOMO and LUMO) on the 2D-CAP/R, considering R = NH_2_ and R = CN. It is worth noting that both the FMOs are predominantly delocalized over the horizontal segments (edges) of the 2D-CAP/NH_2_; however, the HOMO exhibits greater localization around the substituent. Similarly, for 2D-CAP/CN, the HOMO is delocalized over the vertical segments, while the LUMO extends mostly over the left horizontal one (left edge), with a greater concentration around the substituent. These results help clarify which types of analytes are more likely to interact effectively with specific regions of the COFs: (i) electron-accepting species are expected to interact with regions dominated by the HOMO, whereas (ii) electron-donating species should preferentially interact with the LUMO (which are consistent with the CAFI data).

**Figure 10:**
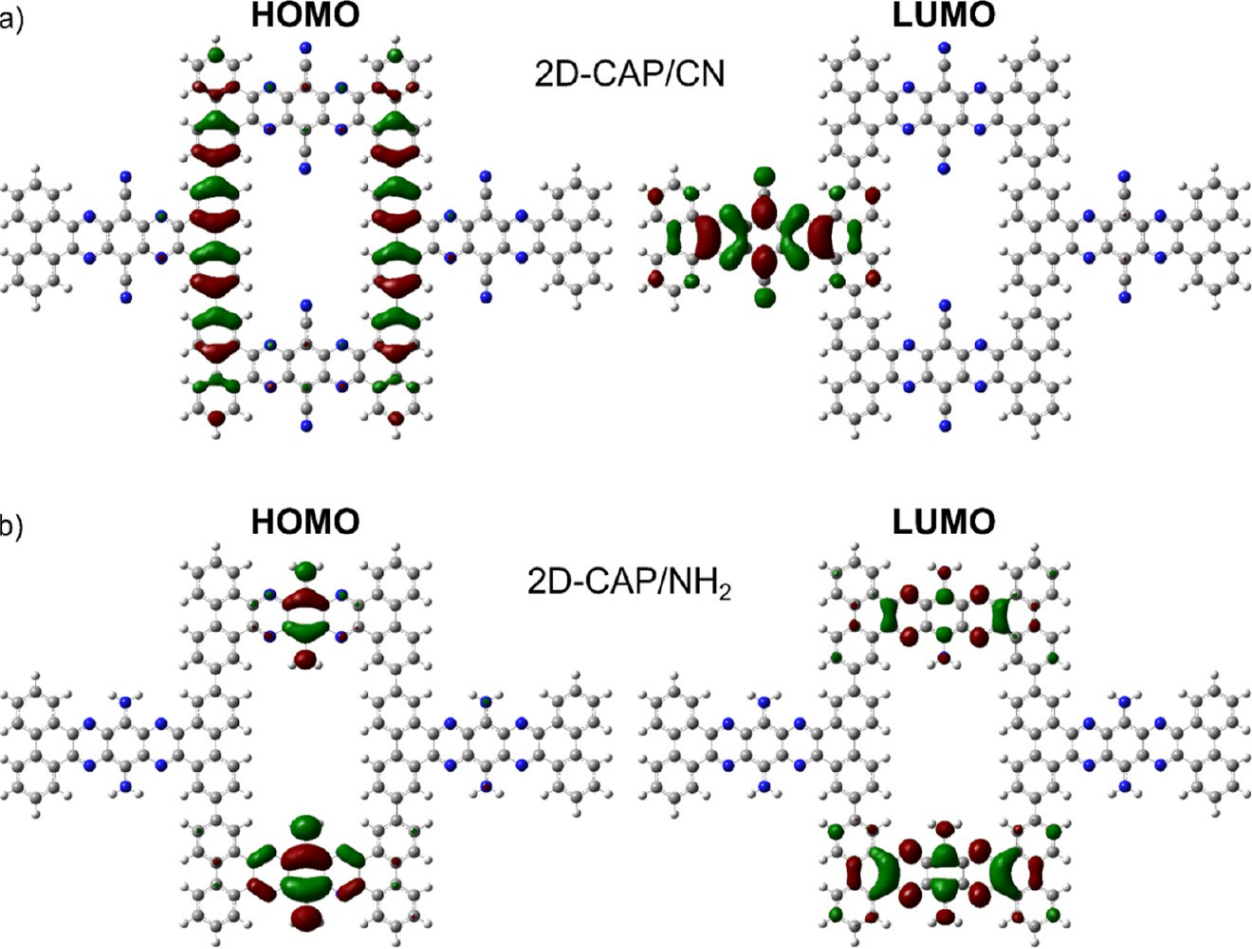
Spatial distribution of the KS-FMOs (HOMO and LUMO) for (a) 2D-CAP/CN_2_ and (b) 2D-CAP/NH_2_ systems.

To better evaluate the changes induced by side groups on the electronic structure of TBQP-COF, the density of electronic states around the Frontier levels of the 2D-CAP system was conducted. Figure 11 shows a comparative analysis of the total density of states (TDOS) of nonmodified 2D-CAP (black line) and modified 2D-CAP/R structures in relation to the partial density of states (PDOS) projected on the side groups (green lines), on the main core of 2D-CAP (blue lines), and the TDOS of the resulting system (2D-CAP/R: red lines). The red and black arrows (dashed lines) indicate the positions of HOMO and LUMO of the unmodified 2D-CAP/H, respectively, while the arrows indicate the FMOs of modified systems, 2D-CAP/R.

**Figure 11:**
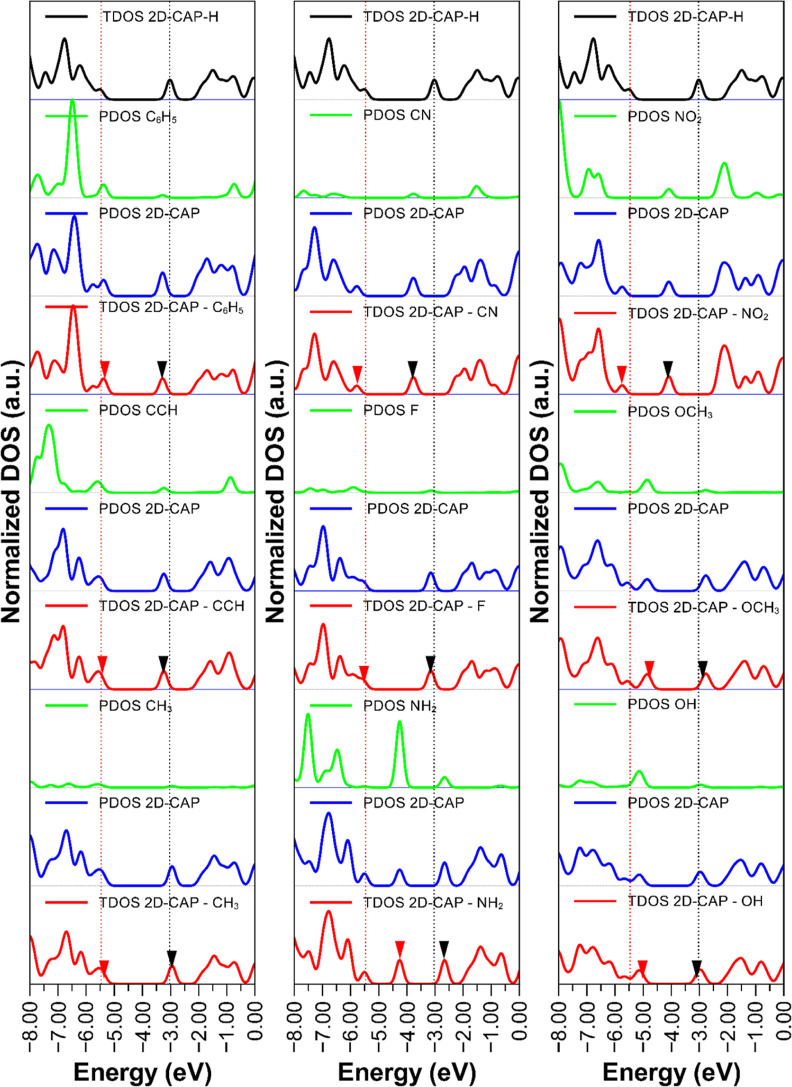
Comparative analysis of the TDOS of nonmodified (2D-CAP/H: black lines) and modified 2D-CAP (2D-CAP/R: red lines) around the Frontier levels. PDOS projected on the side groups (R: green lines) and main core (2D-CAP: blue line) of 2D-CAP/R are also shown.

The unmodified system 2D-CAP/H (or simply 2D-CAP) exhibits *E*_HOMO_ = −5.46 eV and *E*_LUMO_ = −3.02 eV, resulting in an electronic gap of 2.44 eV. In general, three scenarios from chemical modifications are noticed, which affect the electronic gaps in different ways: (i) reduction of the electronic gap due to a decrease in *E*_LUMO_ with small changes on the HOMO (R = CN and NO_2_); (ii) reduction of the electronic gap due to an increase of *E*_HOMO_ with small changes on the LUMO (R = NH_2_, OH, and OCH_3_); and (iii) small changes on the electronic gap (R = C_6_H_5_, CCH, CH_3_, and F). It is worth reiterating the significant alterations induced by the presence of the CN, NH_2_, and NO_2_ side groups.

### 4.3 Adsorption Studies: Influence of Analytes on the Electronic Structure of TBQP-COF Derivatives

To evaluate the effects induced by external chemical species on TBQP-COF derivatives, adsorption studies were conducted for selected analytes: Cl_2_, ClF_3_, and SO_2_ on distinct regions of 2D-CAP, 2D-CAP/CN, and 2D-CAP/NH_2_ (inner/outer edge or vertex). Such chemical species have been chosen based on the relative alignment between their FMOs and the constituent blocks of the TBQP-COF.

The adsorption centers have been selected according to the CAFI results for isolated 2D-CAP/R systems: (i) for R = H: edge or vertex (corner unit); (ii) for R = CN and NH_2_: inner and outer edge units. In fact, molecular sites with elevated CAFI values are expected to play a dominant role in host–guest interactions, acting as preferential regions for charge transfer, charge trapping, exciton quenching, or local electronic doping upon analyte adsorption.

As reported by Liu et al., TBQP preferentially adopts an eclipsed stacking arrangement, so that pore channels are supposed to remain aligned along the crystallographic *c*-axis, preserving the accessibility of the functionalized pore edges to guest analytes.^3^ Although interlayer stacking may influence the overall optoelectronic response of the bulk material, the present study focuses on localized interactions occurring on accessible pore-edge sites or surface-exposed aromatic rings.

Table 3 illustrates the binding energies and recovery times estimated for the adsorbed systems considering distinct adsorption regions. The initial and final structures (after optimization) are presented in the Supporting Information.

**Table 3:** Adsorption Energies and Recovery Times Estimated for the Adsorbed Systems

				recovery times (μs)		
COF	analyte	adsorption region	*E*_ads_ (kcal/mol)	300	400	500
2D-CAP	Cl_2_	vertex	–5.73	1.51 × 10^–2^	1.36 × 10^–3^	3.21 × 10^–4^
		edge	–6.81	9.18 × 10^–2^	5.27 × 10^–3^	9.50 × 10^–4^
	ClF_3_	vertex	–2.90	1.29 × 10^–4^	3.82 × 10^–5^	1.84 × 10^–5^
		edge	–2.31	4.82 × 10^–5^	1.83 × 10^–5^	1.02 × 10^–5^
	SO_2_	vertex	–5.22	6.35 × 10^–3^	7.11 × 10^–4^	1.91 × 10^–4^
		edge	–6.01	2.40 × 10^–2^	1.93 × 10^–3^	4.24 × 10^–4^
2D-CAP/CN	Cl_2_	inner edge	–5.21	6.21 × 10^–3^	6.99 × 10^–4^	1.89 × 10^–4^
		outer edge	–4.88	3.58 × 10^–3^	4.63 × 10^–4^	1.36 × 10^–4^
	ClF_3_	inner edge	–1.27	8.36 × 10^–6^	4.92 × 10^–6^	3.58 × 10^–6^
		outer edge	–3.17	2.03 × 10^–4^	5.38 × 10^–5^	2.42 × 10^–5^
	SO_2_	inner edge	–4.79	3.06 × 10^–3^	4.12 × 10^–4^	1.24 × 10^–4^
		outer edge	–5.25	6.65 × 10^–3^	7.37 × 10^–4^	1.97 × 10^–4^
2D-CAP/NH2	Cl_2_	inner edge	–25.00	1.63 × 10^12^	4.56 × 10^7^	8.46 × 10^4^
		outer edge	–24.44	6.40 × 10^11^	2.26 × 10^7^	4.83 × 10^4^
	ClF_3_	inner edge	–5.94	2.12 × 10^–2^	1.76 × 10^–3^	3.94 × 10^–4^
		outer edge	–3.79	5.78 × 10^–4^	1.18 × 10^–4^	4.54 × 10^–5^
	SO_2_	inner edge	–8.99	3.56	0.08	8.53 × 10^–3^
		outer edge	–9.22	5.21	0.11	0.01

The *E*_ads_ values reveal three distinct interaction regimes: (i) weak adsorption is observed for ClF_3_ (−1 to −3 kcal/mol), indicating highly reversible physisorption; (ii) moderate adsorption energies (−4 to −7 kcal/mol), observed for most Cl_2_ and SO_2_ systems, indicating stable but reversible host–guest interactions, desirable for reusable sensing devices; and (iii) very strong adsorption energies for NH_2_-functionalized systems + Cl_2_ exhibit (∼−25 kcal/mol), suggesting substantial charge-transfer contributions and partial chemisorption character. Although such strong interactions may enhance sensitivity, the associated long recovery times indicate potential limitations for reversible sensing applications. The recovery times corroborate these interaction regimes. In particular, the very strong adsorption observed for 2D-CAP/NH_2_+Cl_2_ systems evidence its limited reversibility.

Figure 12 compares the density of states (DOS) around the Frontier energy levels for isolated 2D-CAP/R (black lines) in relation to components of the adsorbed systems (partial DOS, i.e., PDOS, blue: analyte, green: 2D-CAP/R) and the overall adsorbed systems (red lines) for the distinct analytes (Cl_2_, ClF_3_, and SO_2_) and distinct adsorption centers (inner/outer edge or vertex).

**Figure 12:**
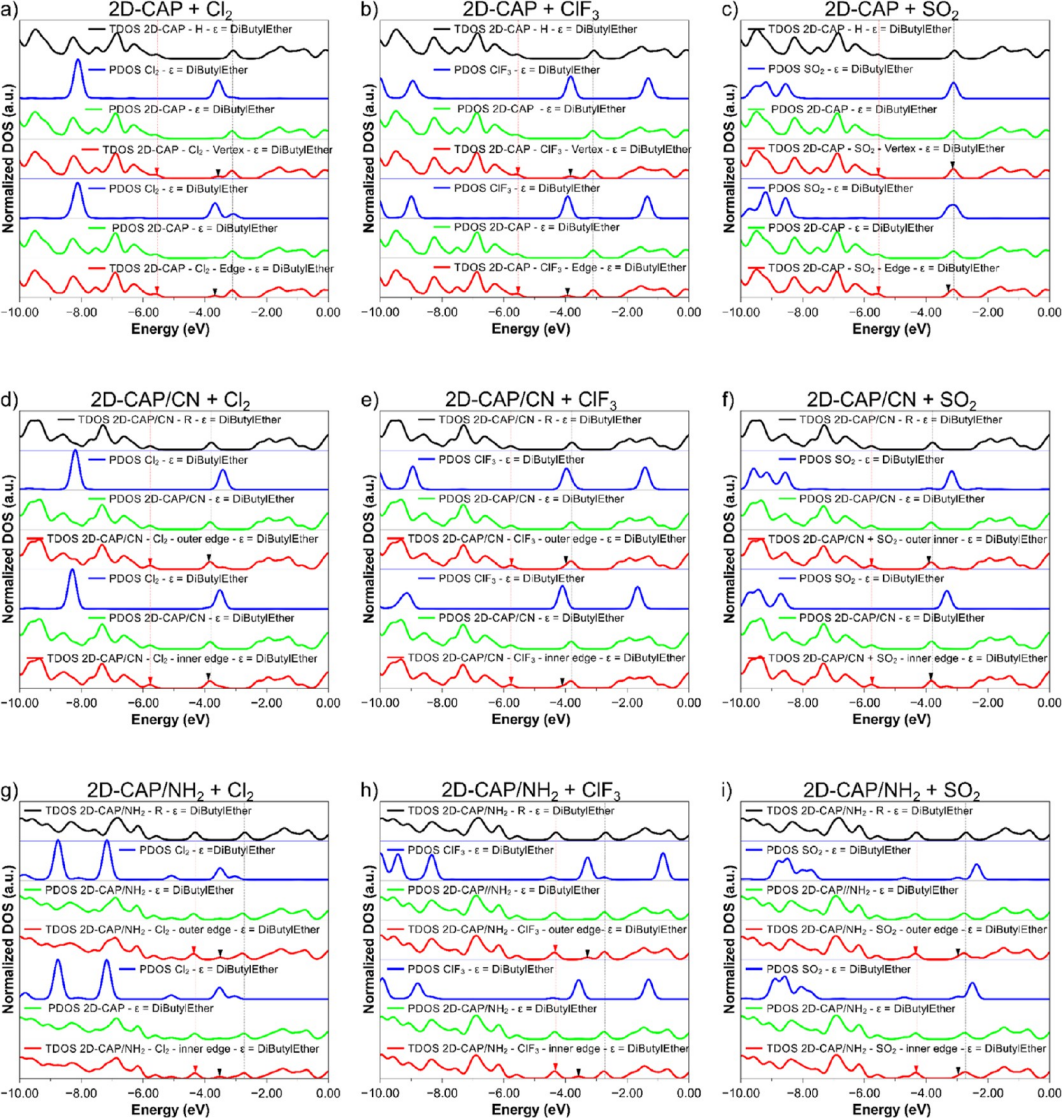
DOS of nonadsorbed and adsorbed 2D-CAP/R systems (R = H, CN, NH_2_) considering the analytes: Cl_2_ (a,d,g), ClF_3_ (b,e,h), and SO_2_ (c,f,i).

Note that the effects of adsorption at the edge and vertex are similar for all the systems. In particular, a new unoccupied state emerges within the band gap in two situations: (i) near the LUMO for 2D-CAP adsorbed with the three analytes, 2D-CAP/CN adsorbed with Cl_2_ and ClF_3_, and 2D-CAP/NH_2_ adsorbed with SO_2_ (very small for 2D-CAP + SO_2_, 2D-CAP/CN + ClF_3_); and (ii) around the middle of the gap for 2D-CAP/NH_2_ adsorbed with Cl_2_ and ClF_3_. Such changes can influence electron transport processes and promote effective photoluminescence quenching by photoinduced electron transfer mechanisms. Minimal or no significant effects are expected in cases where the analyte’s LUMO is higher than COF, as observed for 2D-CAP/CN + Cl_2_ and 2D-CAP/CN + SO_2_.

In this context, the 2D-CAP and 2D-CAP/NH_2_ structures demonstrate promising potential for detecting Cl_2_ and ClF_3_, although with reduced effectiveness for SO_2_. In contrast, 2D-CAP/CN is expected to exhibit a comparatively lower sensing performance. Given the relative alignment of the FMOs, the analytes tend to act as electron traps in the TBQP-COFs.

The comparative analysis of analyte positioning on the inner edge (near the COF pore) versus the outer edge reveals negligible variations across all of the cases, supporting the validity of employing reduced models as reliable representations of COF structures.

Aiming to evaluate the plausibility of optical responses in the systems, the optical absorption spectra of the adsorbed systems were evaluated. Although extended π-conjugated COFs may exhibit H-/J-type stacking and interlayer excitonic coupling that contributes to the baseline optical properties of the material, the present analysis focuses on localized host–guest interactions. In this sense, the fragment-based approach adopted here is expected to capture relative electronic perturbations induced by analyte adsorption, despite possible quantitative differences in the absolute optical response of the bulk material. Figure 13 shows the theoretical adsorption spectra associated with 2D-CAP/R derivatives, before and after analytes’ adsorptions.

**Figure 13:**
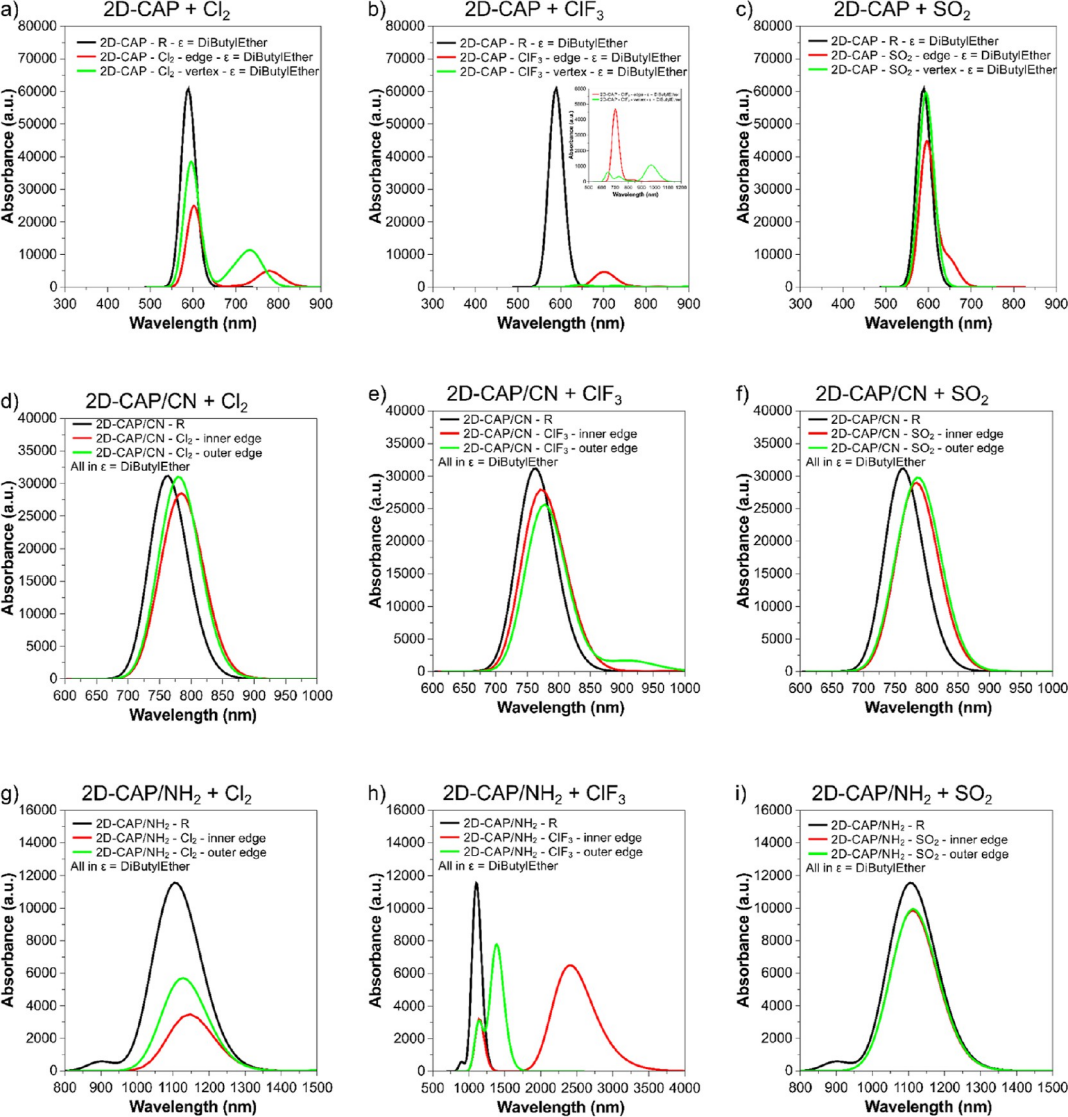
Optical absorption spectra of isolated and absorbed 2D-CAP, 2D-CAP/CN, and 2D-CAP/NH_2_ systems considering the gaseous analytes: Cl_2_ (a,d,g), ClF_3_ (b,e,h), and SO_2_ (c,f,i).

In the unmodified 2D-CAP, Cl_2_ adsorption leads to significant amplitude changes in the optical absorption spectrum. Notably, the appearance of a secondary peak (∼750–780 nm) may serve as a marker for chlorine detection. Smaller spectral changes are noticed for SO_2_, with an additional peak observed only when the analyte is located at the edge. Finally, the presence of ClF_3_ induces the formation of a number of intermediate transition states, hindering the evaluation of the changes induced on the main peak (even for five singlet transitions in TD-DFT calculations).

In the 2D-CAP/CN derivative (Figure 12d–f), a strong ∼758 nm peak characterizes the isolated structure. This peak undergoes only minimal bathochromic shifts and intensity variations upon analyte adsorption at any site. Consequently, although 2D-CAP/CN demonstrates gas-sensing capability, its optical response is largely nonselective across different analytes.

For the 2D-CAP/NH_2_ derivative (Figure 12g–i), the isolated structure exhibits prominent low-energy transitions (near-infrared range) with absorption peaks near 900 and 1100 nm. Cl_2_ adsorption attenuates these peaks and induces a slight redshift. Conversely, ClF_3_ adsorption triggers substantial spectral modifications for both interaction sites, characterized by pronounced intensity quenching, peak shifts, and the emergence of new secondary peaks at ∼1141 and 1147 nm. In stark contrast, SO_2_ adsorption leads to only negligible spectral changes, regardless of the interaction site. This marked differential optical response demonstrates the strong potential and intrinsic selectivity of 2D-CAP/NH_2_ for the optical detection of halogen-based analytes (Cl_2_, ClF_3_) over SO_2_.

Therefore, the DFT-based study establishes that targeted edge functionalization of TBQP-COFs (specifically with –NH_2_ groups) provides a rational and effective strategy to engineer both sensitivity and selectivity in optical chemical sensing, directly linking tunable electronic structure to analyte-specific optoelectronic responses of such porous sensory materials.

### 4.4 Full Atomistic Reactive Molecular Dynamics

To evaluate the adsorption capabilities of the selected derivatives 2D-CAP/R (for R = H, CN and NH_2_), fully atomistic reactive molecular dynamics (FARMD) simulations were performed. The simulation employed a triclinic simulation box with dimensions of 25 × 17 × 50 Å^3^, ensuring periodic boundary conditions (PBCs). Specifically, a single, isolated COF layer was considered and positioned near the center along the *Z*-axis, with a large vacuum region (within the 50 Å *z*-dimension) to prevent unphysical interactions between periodic images along the stacking direction. This single-layer approach was deliberately chosen to isolate the primary, in-plane host–guest interactions within the pore, allowing us to evaluate primary binding preferences unhindered by interlayer diffusion barriers. The COF fragment was positioned near the center along the *Z*-axis, with its lateral edges placed close to the box boundaries, allowing the PBC to reconstruct the extended COF structure. Only Cl_2_ and SO_2_ were simulated, given the availability of ReaxFF parameters.^50^

Figure 14 illustrates the rectangular base of the simulation box and the positioning of the 2D-CAP fragment within it. Figure 15 illustrates color maps associated with average molecular distances between 2D-CAP/R and analytes along the FARMD simulations. Red and blue colors indicate, respectively, the closest and farthest interactions considering all of the production steps of the simulations. The color scales were projected on selected snapshots (for a minimum 2D-CAP/R-analyte distance). Radial distribution function (g­(r)) analyses are presented in the Supporting Information.

**Figure 14:**
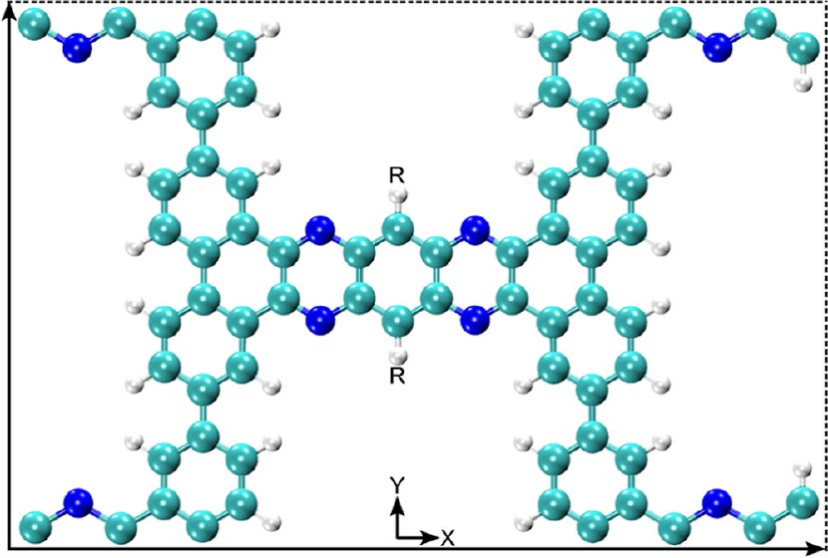
Rectangle simulation box base and 2D-CAP/R positioning. Carbon is cyan, nitrogen is blue, and hydrogen is white.

**Figure 15:**
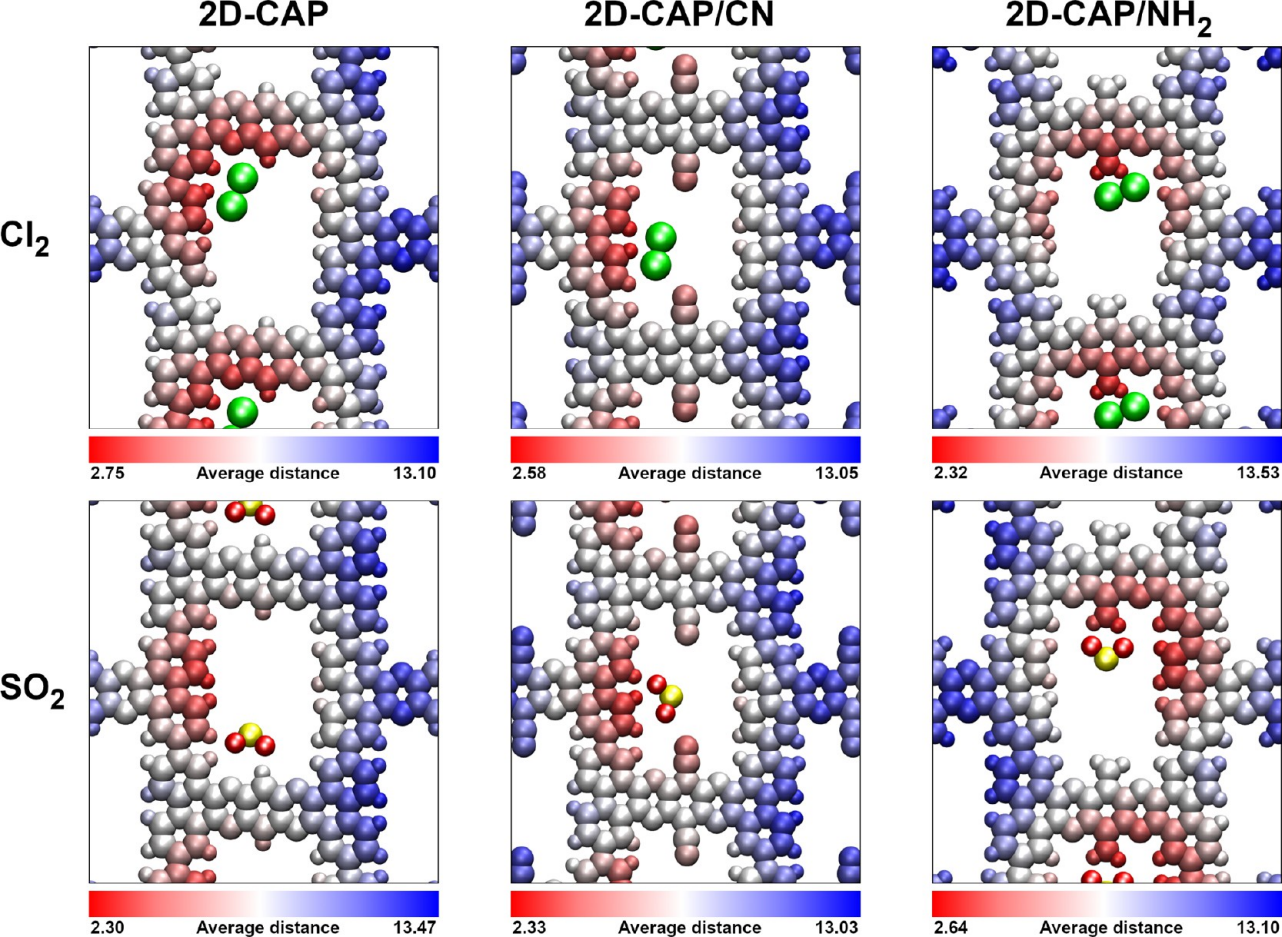
2D-CAP/R-analytes average molecular distances estimated along the FARMD simulation projected on 2D-CAP/R structures (snapshots associated with the minimum distances).

It is observed that the Cl_2_ and SO_2_ analytes tend to strongly adsorb onto the hydrogen atoms located at the corners of the 2D-CAP and 2D-CAP/NH_2_ pores, as well as onto the hydrogen atoms positioned along the pore edges of 2D-CAP/CN. Notably, the dynamics reveal that the interactions occur primarily through these in-plane hydrogen atoms and functional groups, rather than via direct π–π interactions with the aromatic rings. In particular, the oxygen atoms of SO_2_ preferentially interact with the hydrogen atoms located along the lateral edges of the 2D-CAP pore. This behavior can be attributed to the presence of a region of lower electrostatic potential around the oxygen atoms of SO_2_, which promotes interaction with regions of higher positive potential in the COF (see Supporting Information). In the case of 2D-CAP/CN, a region of higher electrostatic potential appears to be located along the inner pore edge, leading to a stronger attraction of SO_2_ toward the internal region of the pore. This suggests a more pronounced preferential adsorption of SO_2_ inside the pore of 2D-CAP/CN. Notably, these COFs are effective in adsorbing analytes even under minimal concentration conditions (around 1 atm).

Note that 2D-CAP interacts effectively with both analytes. The adsorptions take place at the inner region of the COF, and the interactions occur primarily within the plane of the COF structure. Cl_2_ tends to interact with the inner corner of the COF, while SO_2_ adsorbs preferentially around the hydrogen atoms of the vertical segment.

Very similar results are observed for 2D-CAP/CN; however, the presence of the side group drives the interactions to the hydrogen atoms of the vertical segment for both the analytes. The effective role of the substituent is noticed for 2D-CAP/NH_2_. In these systems, the interaction is driven toward the R groups. Moreover, given their high *f* ^–^ values for –NH_2_, it could suggest that it could act as an effective electron donation center.

In summary, the FARMD simulations indicate relevant analyte adsorption capabilities for all of the systems. It is noticed that the dominance of electrostatic effects on the analyte adsorption, in general, oxygen/chlorine atoms of the analytes, tends to interact with positively charged hydrogen atoms (from corner units). An effective direct effect of substituents is noticed for 2D-CAP/NH_2_. By analyzing the FARMD results in conjunction with CAFIs (Figure 3) it is possible to estimate an effective intermolecular interaction for 2D-CAP + Cl_2_, 2D-CAP/NH_2_+Cl_2_, and 2D-CAP/NH_2_+SO_2_ once in these systems the adsorption centers are close to very reactive sites, suggesting the plausibility of charge-transfer processes. Such effects could lead to measurable changes in electrical (charge carrier trapping/doping) and optical properties (fluorescence quenching by photoinduced electron transfer), highlighting their relevance as chemical sensors.

## 5 Conclusions

This study explored the electronic structure and reactivity of TBQP-based COFs and selected functionalized derivatives, with the aim of assessing their potential as chemical sensors for toxic gases, e.g., Cl_2_, ClF_3_, and SO_2_.

The analysis of energy levels revealed that the optoelectronic properties of unmodified COFs are primarily governed by the corner (HOMO) and edge (LUMO) subunits, highlighting the role of gap engineering as an effective strategy for tuning the electronic behavior of these materials.

Local chemical reactivity and adsorption analyses showed that specific substitutions at the central edge, particularly with –NH_2_, enhance the local reactivity and promote stronger interactions with analyte molecules. The presence of analytes around this COF induces the formation of new (empty) electronic states within the band gap, which can lead to significant changes in electrical and optical properties of the systems, confirming their suitability for sensing applications.

In general, FARMD simulations supported the CAFI-predicted reactive sites (mainly for R = NH_2_), validating the use of molecular dynamics to complement quantum approaches in larger-scale systems.

Altogether, the results confirm the potential of TBQP-based COFs as versatile platforms for chemical sensing. They also highlight the importance of integrating quantum and classical simulations to guide the rational design of high-performance, selective, and tunable sensor materials for environmental and industrial applications.

## Supplementary Material



## Notes

Additional data regarding the saturation of electronic properties with structure size; local reactivity of extended structures; additional information regarding Frontier molecular orbitals and electrostatic potential; and additional data for adsorption studies and FARMD simulations, including *g*(*r*) analyses (PDF)
