# Supplementary material for: Modulating Optoelectronic Properties of TBQP-Based Covalent Organic Frameworks for Chemical Sensing
Source: ACS Omega. 2026 Jun 16;11(25):37707–23. doi: 10.1021/acsomega.6c02636 (PMC13325357; doi:10.1021/acsomega.6c02636)
Supplement: Supplementary file 1 [file ao6c02636_si_001.pdf]

SUPPORTING INFORMATION

Modulating optoelectronic properties of TBQP-based covalent organic frameworks for chemical sensing

Levy Alvarenga Galindo [1*], Ricardo Paupitz [2], Augusto Batagin-Neto [1,3]

[1] São Paulo State University (UNESP), School of Sciences, POSMAT, Bauru/SP 17033-360, Brazil

[2] Physics Department, São Paulo State University - UNESP, 13506-900, Rio Claro, SP, Brazil

[3] São Paulo State University (UNESP), Institute of Sciences and Engineering, Itapeva/SP 18409-010, Brazil

*Corresponding author: a.batagin@unesp.br

Summary

S1. Saturation of electronic properties as a function of structure size.................................2

S2. Local reactivity of extended structures...3

S3. Frontier Orbitals..5

S4. Electrostatic Potential...7

S5. Additional results for adsorption studies..8

S6. FARMD Results...11

S1. Saturation of electronic properties as a function of structure size

To assess the saturation limit of the optoelectronic properties of TBQP-COF, and thus define a model system that would represent it appropriately, structures containing between two and six repeating units were built from the basic 1D-CAP unit. The same procedure was carried out for the 2D-CAP and 2D-CAP-2 structures, containing between one and five units and between one and three units, respectively (Figure S1). Table S1 and Figure S1 show the energy values of the HOMO (E_{HOMO}) and LUMO (E_{LUMO}) frontier orbitals, as well as the electronic *gaps* (E_{gap}) of the different TBQP-COF extended structures.

Table S1. Some results of the TBQP-COF 1D-CAP, 2D-CAP and 2D-CAP-2 structures**.**

Systems	Nº of Units	E_{HOMO} (eV)	E_{LUMO} (eV)	E_{gap} (eV)
**1D-CAP**	1	-5.83	-2.96	2.88
	2	-5.59	-3.06	2.53
	3	-5.47	-3.05	2.41
	6	-5.41	-3.07	2.34
	5	-5.38	-3.10	2.28
	6	-5.36	-3.09	2.28
**2D-CAP**	1	-5.52	-3.16	2.37
	2	-5.53	-3.16	2.37
	3	-5.55	-3.18	2.37
	4	-5.55	-3.17	2.38
	5	-5.55	-3.19	2.36
**2D-CAP-2**	1	-5.45	-3.16	2.29
	2	-5.47	-3.19	2.28
	3	-5.46	-3.19	2.28

As can be seen, as the number of units increases, the energies tend to reach an approximately constant value. In general, the absolute values of E_{HOMO} and E_{LUMO} are close for the different systems, oscillating around the average values of -5.46 and -3.16 eV, respectively, with more significant variations for 1D-CAP. There is also a tendency for the electronic *gap to* saturate at around 2.28 eV. The saturation trend observed suggests that the reduced structures of COFs can adequately describe the basic electronic characteristics of more extended systems, at a reduced computational cost. In particular, gap values for the 2D-CAP and 2D-CAP-2 systems are almost constant no matter the number of units considered, while for the 1D-CAP systems such saturation only occurs after 4 units.

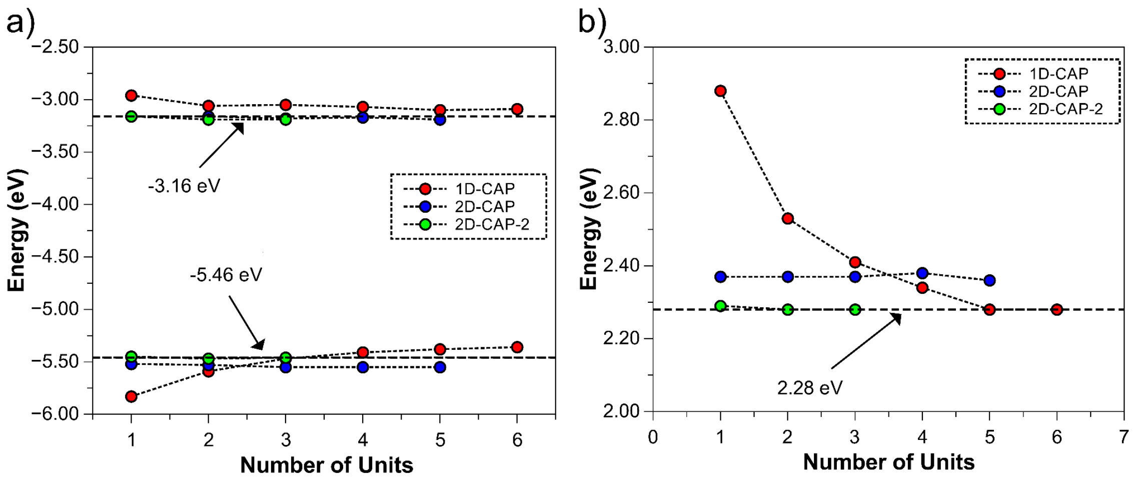

Figure S1. Evolution of: a) E_{HOMO}, E_{LUMO} and b) E_{gap} values with the number of TBQP-COF units.

S2. Local reactivity of extended structures

Figures S2-S3 illustrate the CAFI results obtained for more extended structures of 1D-CAP and 2D-CAP (6 units). The same definitions presented in the main text are employed.

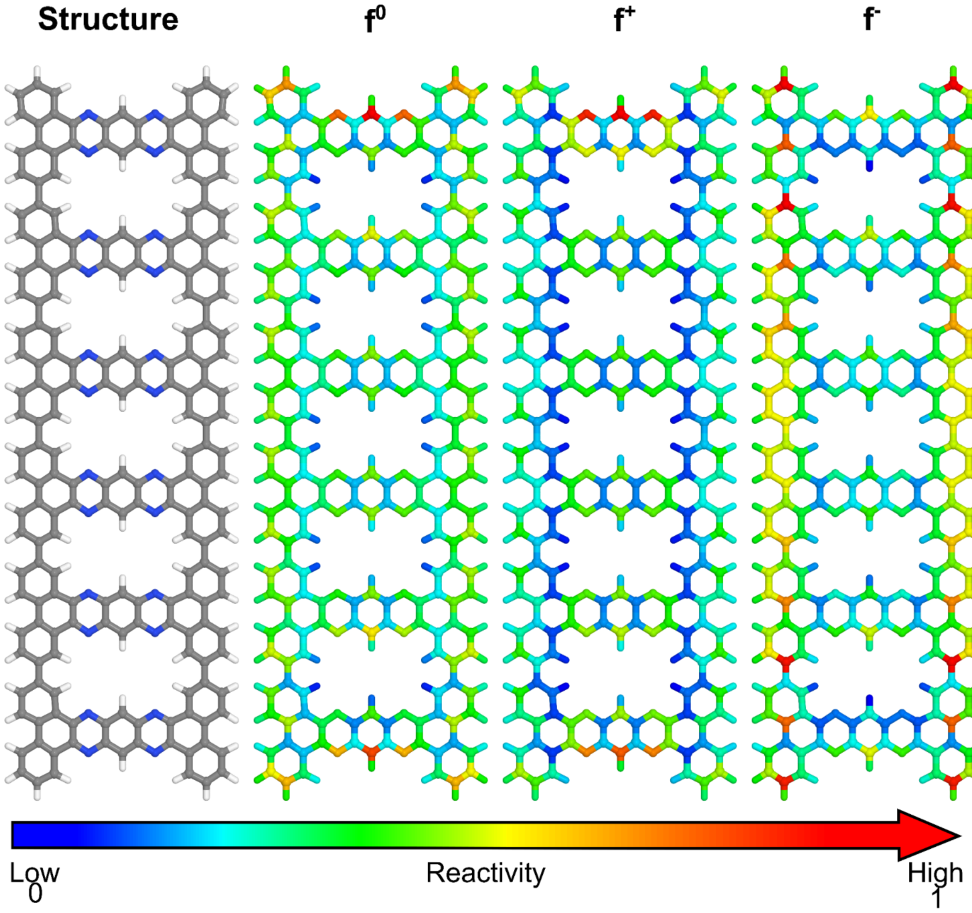

Figure S2. Color maps associated with CAFIs estimated for the extended 1D-CAP system. Atoms in gray, blue and white define carbon, nitrogen and hydrogen, respectively.

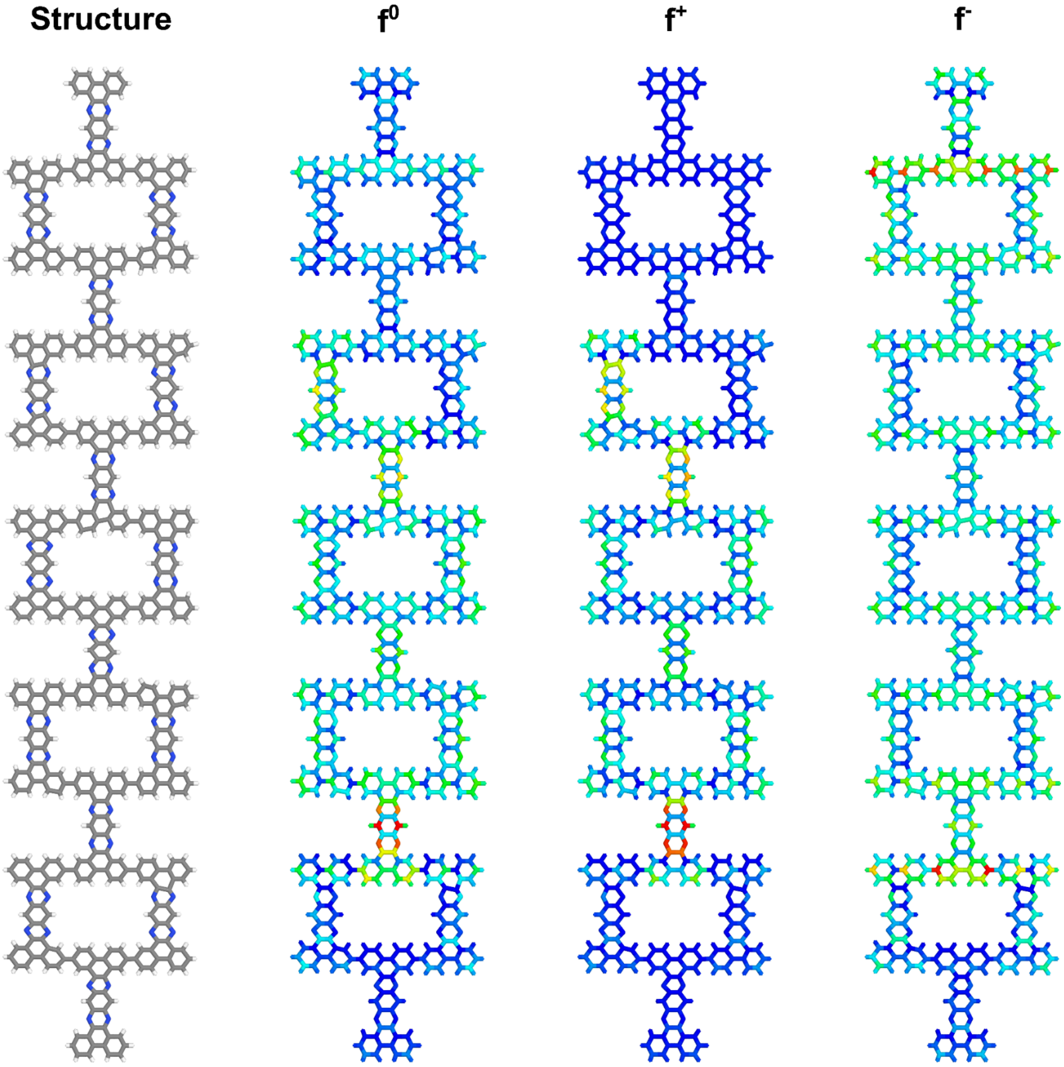

Figure S3. Color maps associated with CAFIs estimated for the extended 2D-CAP system. Atoms in gray, blue and white define carbon, nitrogen and hydrogen, respectively.

S3. Frontier Orbitals

Figure S4 illustrates the spatial distribution of the Kohn-Sham (KS) frontier molecular orbitals (FMO, HOMO, HOMO - 1, HOMO - 2 and LUMO, LUMO +1, LUMO + 2) over the structure of the 2D-CAP.

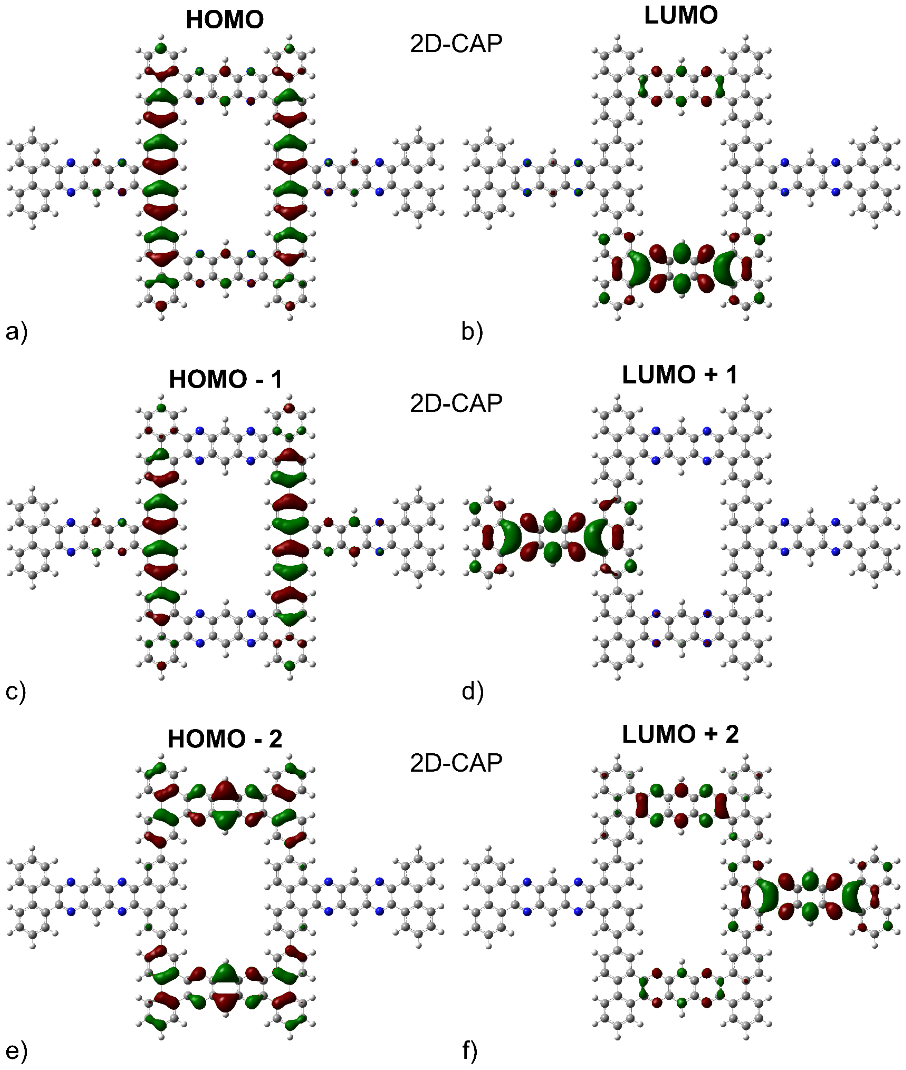

Figure S4. Illustration of the spatial distribution of the FMOs of the 2D-CAP system: a) HOMO, b) LUMO, c) HOMO-1, d) LUMO+1, e) HOMO-2, and f) LUMO+2,

Figure S5 shows the color maps for CAFIs estimated for the TBQP-based COF, considering 2D-CAP-2/R derivatives. Note a set of effects similar to 2D-CAP (see Figure 3) is observed for the 2D-CAP-2 structure, with a greater intensification of the asymmetries. An exception is the centralization of the reactivity associated with f^- for R = CH_3 on the central carbon of the edge, not observed for the 2D-CAP system. It is noted that, in both structures, in general, there is a dominance of the reactivity of the edges with respect to the vertices.

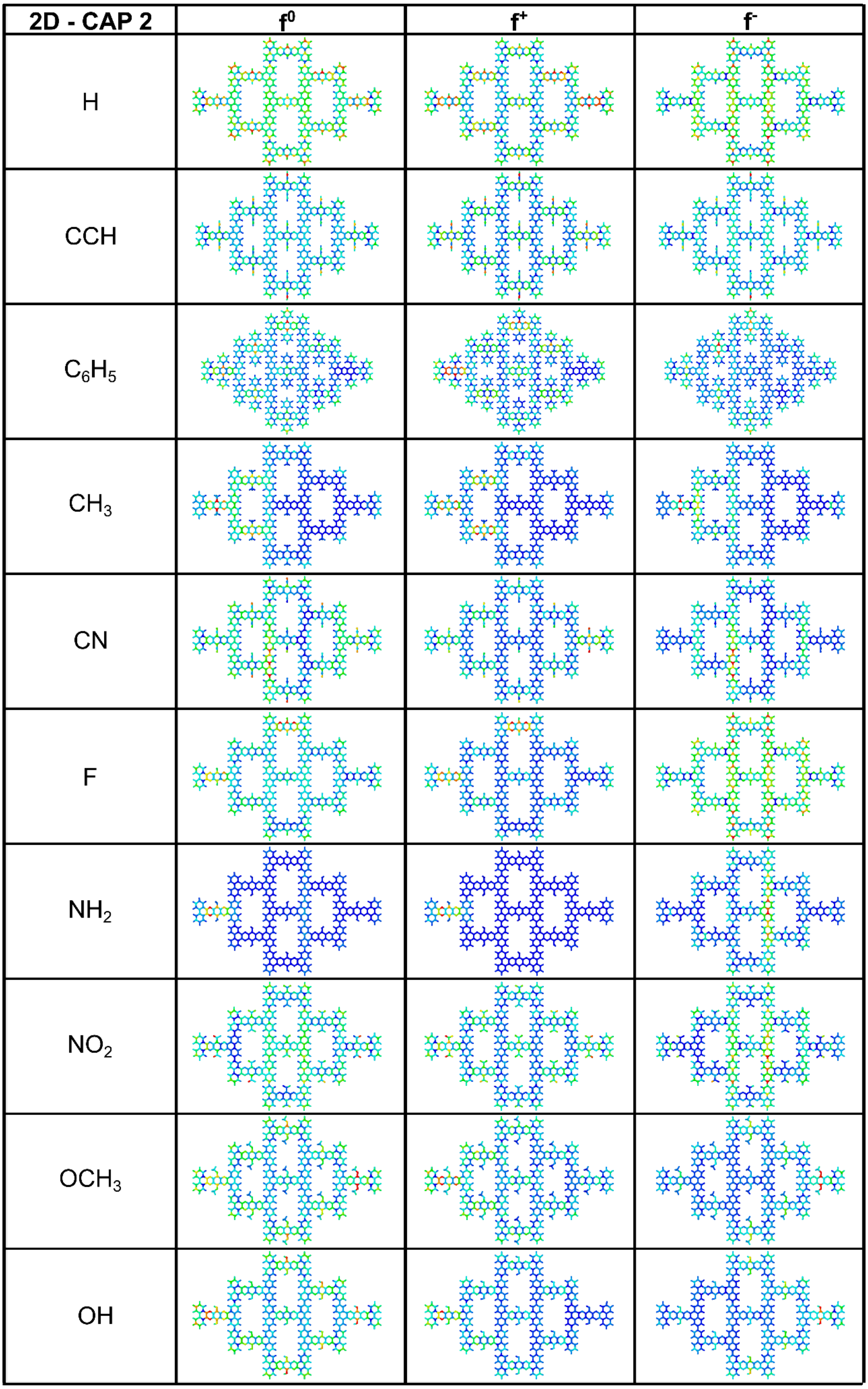

Figure S5. Representation of the CAFI calculated for TBQP-COF 2D-CAP-2 with R = H, CCH, C_6H_5, CH_3, CN, F, NH_2, NO_2, OCH_3 and OH. Red regions indicate more reactive sites with respect to radical (f^0), nucleophilic (f^+) and electrophilic (f^-) species.

S4. Electrostatic Potential

Figure S6 shows the three-dimensional electrostatic potential map estimated for 2D-CAP, 2D-CAP/CN and 2D-CAP/NH_2 (isolated and adsorbed structures for the analytes Cl_2, ClF_3 and SO_2 in dibutyl ether). Numeric values ranging from -2.257×10^{-2} to 2.257×10^{-2} atomic energy units are considered.

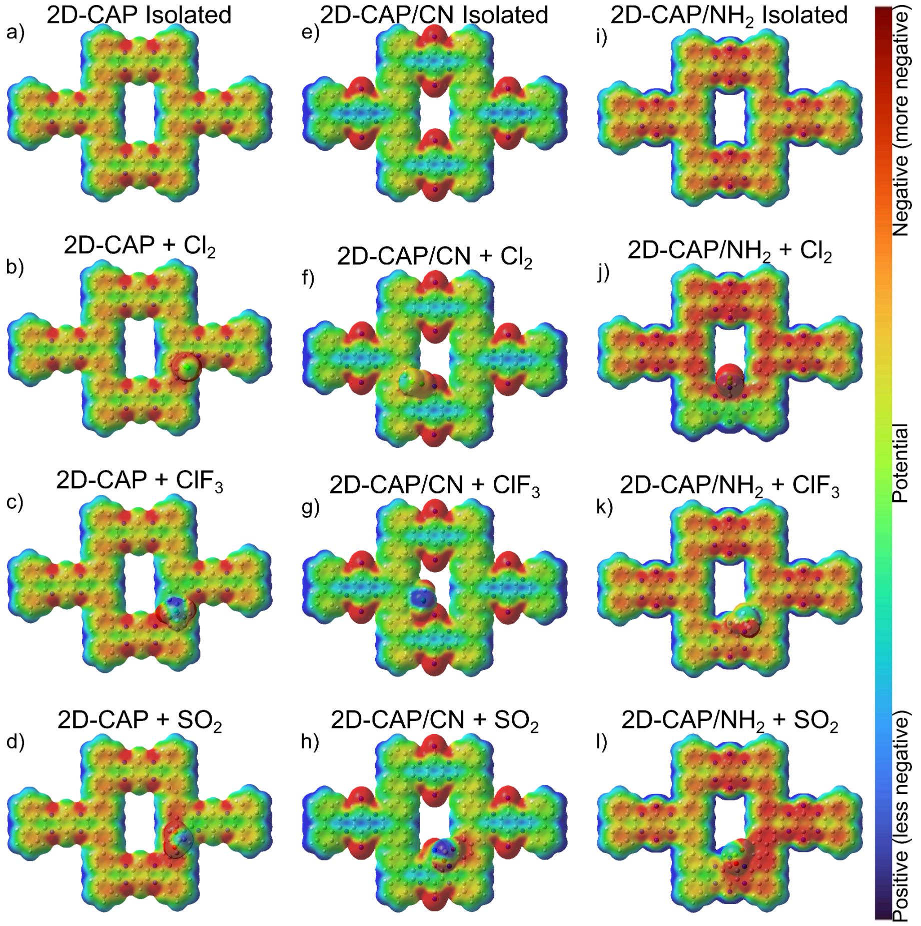

Figure S6. Electrostatic Potential a) 2D-CAP Isolated; b) 2D-CAP + Cl_2; c) 2D-CAP + ClF_3; d) 2D-CAP + SO_2; e) 2D-CAP/CN Isolated; f) 2D-CAP/CN + Cl_2; g) 2D-CAP/CN + ClF_3; g) 2D-CAP/CN + SO_2; i) 2D-CAP/NH_2 Isolated; j) 2D-CAP/NH_2 + Cl_2; k) 2D-CAP/NH_2 + ClF_3; l) 2D-CAP/NH_2 + SO_2 all in dibutyl ether medium.

Note that an increase in electronic density (negatively charged site) is observed in the following regions: i) 2D-CAP: on the nitrogen atoms; ii) 2D-CAP/CN: on the nitrogens of the -CN endings; iii) 2D-CAP/NH_2: distributed throughout the structure, with greater intensity in the region of the Cl_2 analyte and on the sides of ClF_3.

The regions with the highest electrostatic potential (represented in blue), i.e. the lowest electronic density, are observed on the side edges of the 2D-CAP and 2D-CAP/CN structures, in the inner region of the COFs and on the sulfur of SO_2. In addition, it can be

seen that the electronic density is evenly distributed over the 2D-CAP and 2D-CAP/CN structures.

Analysis of the electrostatic map suggests that the analytes Cl_2, ClF_3 and SO_2 tend to be attracted to the inner region of the COFs. In particular, the results indicate that Cl_2 (in 2D-CAP/NH_2) and ClF_3 (in the three COFs analyzed) are adsorbed more strongly in the inner region of the structure. In addition, the adsorption studies confirm a strong interaction of the ClF_3 gas with the 2D-CAP and 2D-CAP/CN structures.

The electrostatic potential measures the charge distribution around the molecule, where: Blue regions (more positive) represent favorable areas for interactions with nucleophilic species (electron acceptors). Red regions (more negative) indicate favorable areas for interactions with electrophilic species (electron donors). The Fukui indices, in turn, are used to identify regions of greater susceptibility to chemical reactions: f^+ (nucleophilic): indicates sites prone to accepting electrons; f^- (electrophilic): indicates sites prone to donating electrons; f^0 represents the general reactivity of the site.

For an expected correlation between these properties, regions with high f^- (electrophilic) should coincide with red regions in the electrostatic potential (high electron density), while regions with high f^+ (nucleophilic) should coincide with blue regions in the electrostatic potential (low electronic density). However, for the three COFs analyzed (2D-CAP, 2D-CAP/CN and 2D-CAP/NH_2), it was observed that the Fukui index and the electrostatic potential do not always point to the same regions of reactivity. This discrepancy can be attributed to resonance effects, long-range interactions or the need to refine the calculation methodology. Therefore, a complementary analysis, considering the influence of the solvating medium and possible electronic effects of the system, may be necessary for a more accurate interpretation of the results.

S5. Additional results for adsorption studies

Figures S7-S9 present the 2D-CAP, 2D-CAP/CN and 2D-CAP/NH_2 systems in the presence of the three analytes (Cl_2, ClF_3, and SO_2) in their initial (pre-optimization) and final (post-optimization) positions.

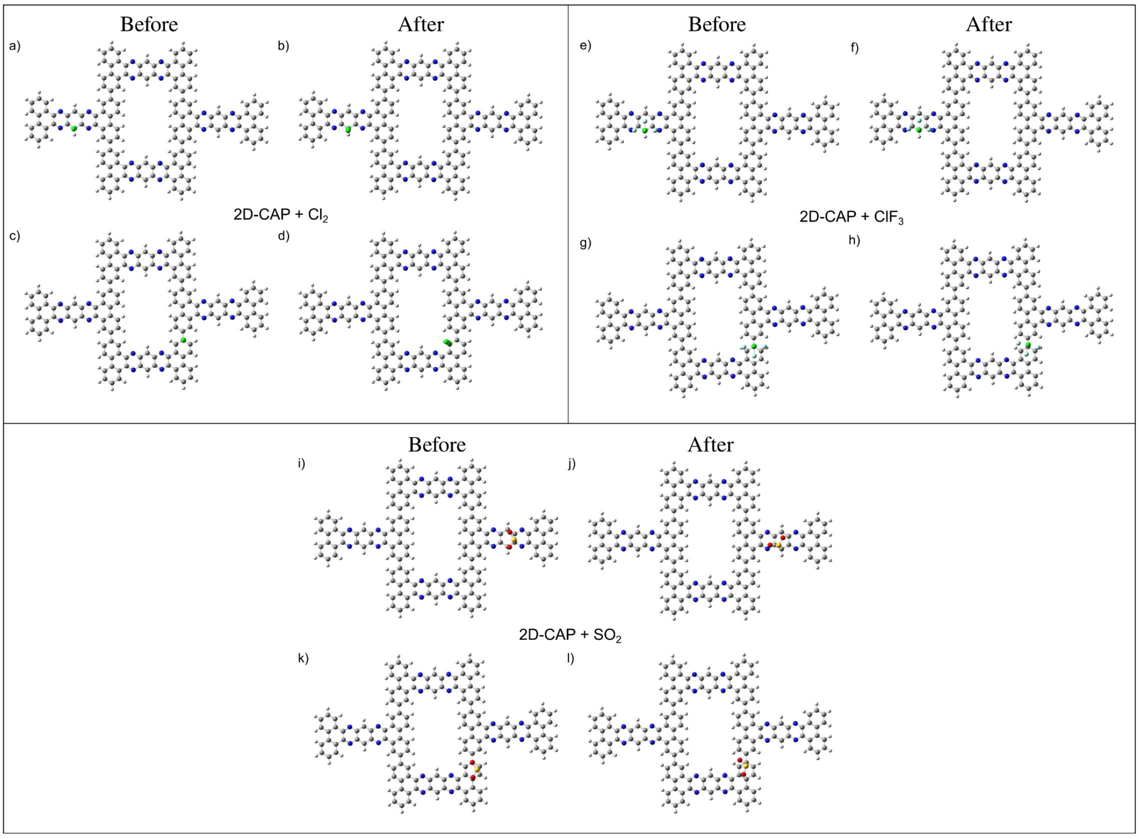

Figure S7. Representation of adsorbed 2D-CAP systems before (a, c, e, g, i, k) and after geometry optimization (b, d, f, h, j, l).

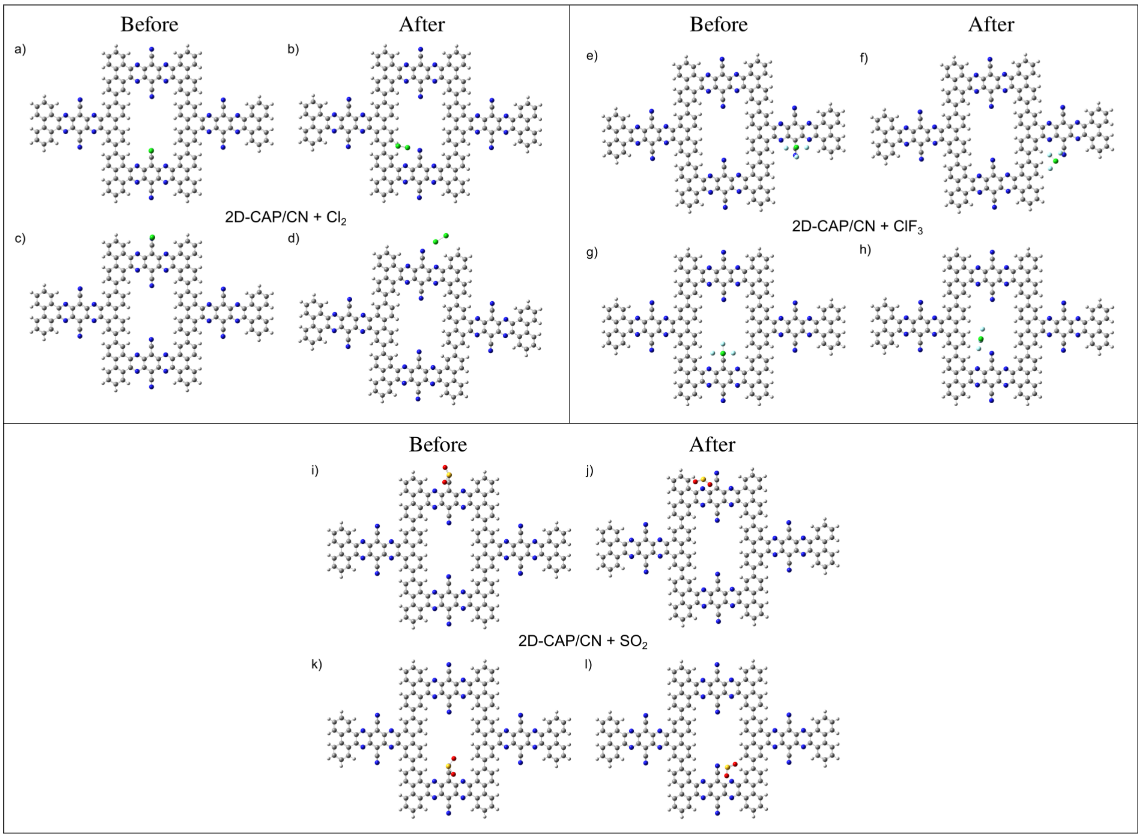

Figure S8. Representation of adsorbed 2D-CAP/CN systems before (a, c, e, g, i, k) and after geometry optimization (b, d, f, h, j, l)

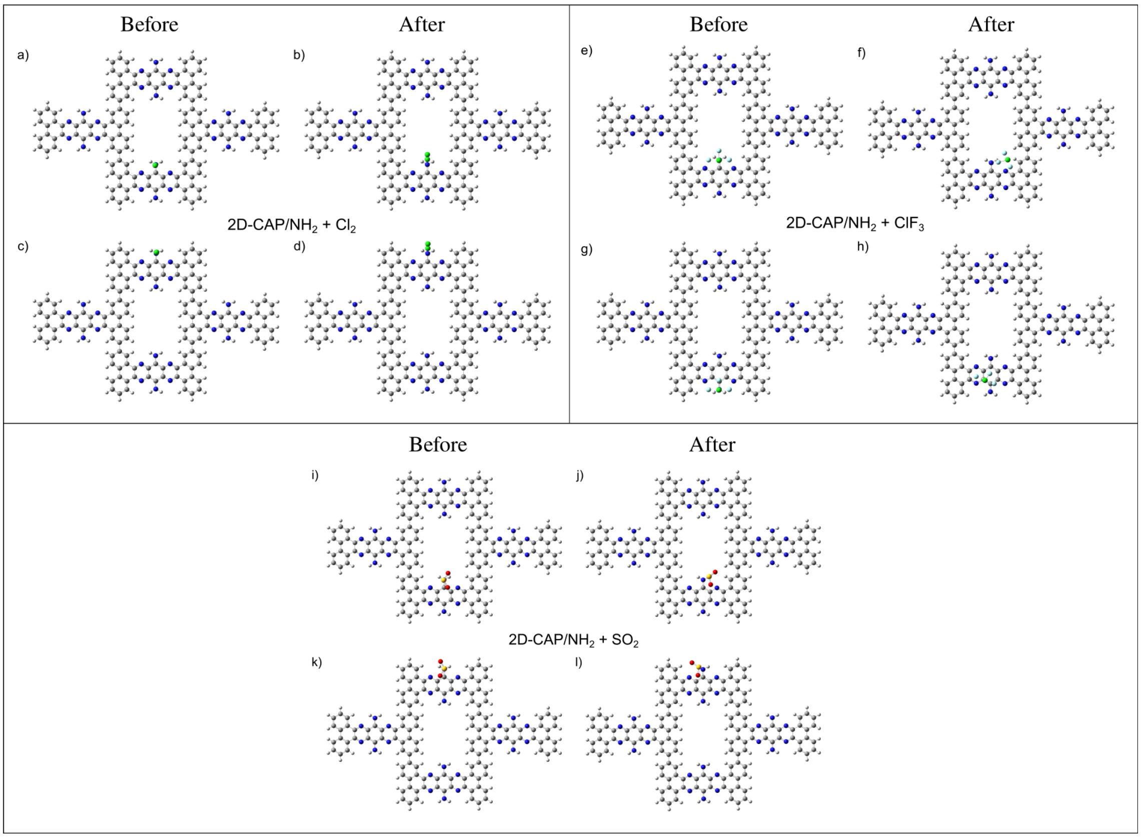

Figure S9. Representation of adsorbed 2D-CAP/NH_2 systems before (a, c, e, g, i, k) and after geometry optimization (b, d, f, h, j, l)

S6. FARMD Results

Figures S10 and S11 show the temperature evolution as a function of simulation steps during the equilibration stage for the distinct systems. All simulations were performed at 300 K using the Nose–Hoover thermostat.

Owing to the limited number of particles, temperature fluctuations of approximately 100 K were observed during the entire equilibration process. To ensure proper system stabilization, the equilibration time was extended to 100,000 fs. Despite these temperature oscillations under the NVT ensemble, the system ultimately achieved a stable equilibrium state after a sufficiently long simulation period.

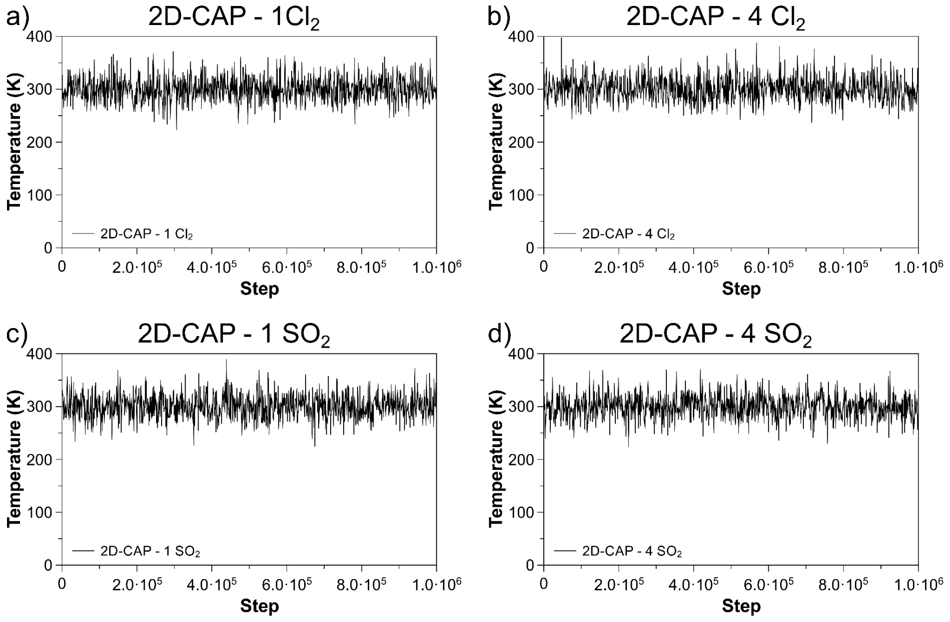

Figure S10. Temperature by steps a) 2D-CAP + 1 Cl_2, b) 2D-CAP + 4 Cl_2, c) 2D-CAP + 1 SO_2, d) 2D-CAP + 4 SO_2.

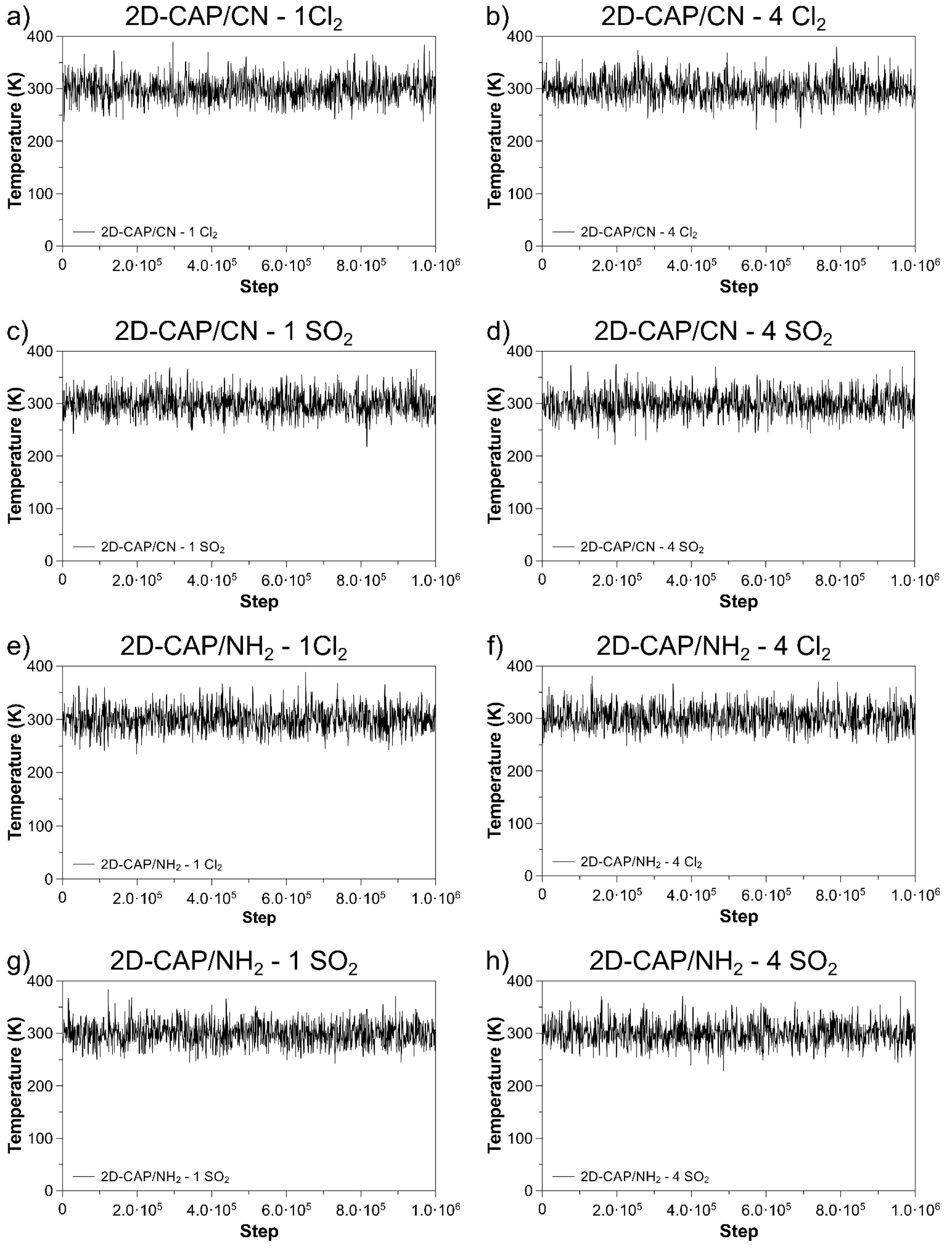

Figure S11. Temperature by steps a) 2D-CAP/CN + 1 Cl_2, b) 2D-CAP/CN + 4 Cl_2, c) 2D-CAP/CN + 1 SO_2, d) 2D-CAP/CN + 4 SO_2; e) 2D-CAP/NH_2 + 1 Cl_2, f) 2D-CAP/NH_2 + 4 Cl_2, g) 2D-CAP/NH_2 + 1 SO_2, h) 2D-CAP/NH_2 + 4 SO_2.

Figures S12-S14 bring the FARMD results for 2D-CAP, 2D-CAP/CN and 2D-CAP/NH_2, respectively. It is observed that the Cl_2 and SO_2 analytes tend to strongly adsorb onto the hydrogen atoms located at the corners of the 2D-CAP and 2D-CAP/NH_2

pores, as well as onto the hydrogen atoms positioned along the pore edges of 2D-CAP/CN.

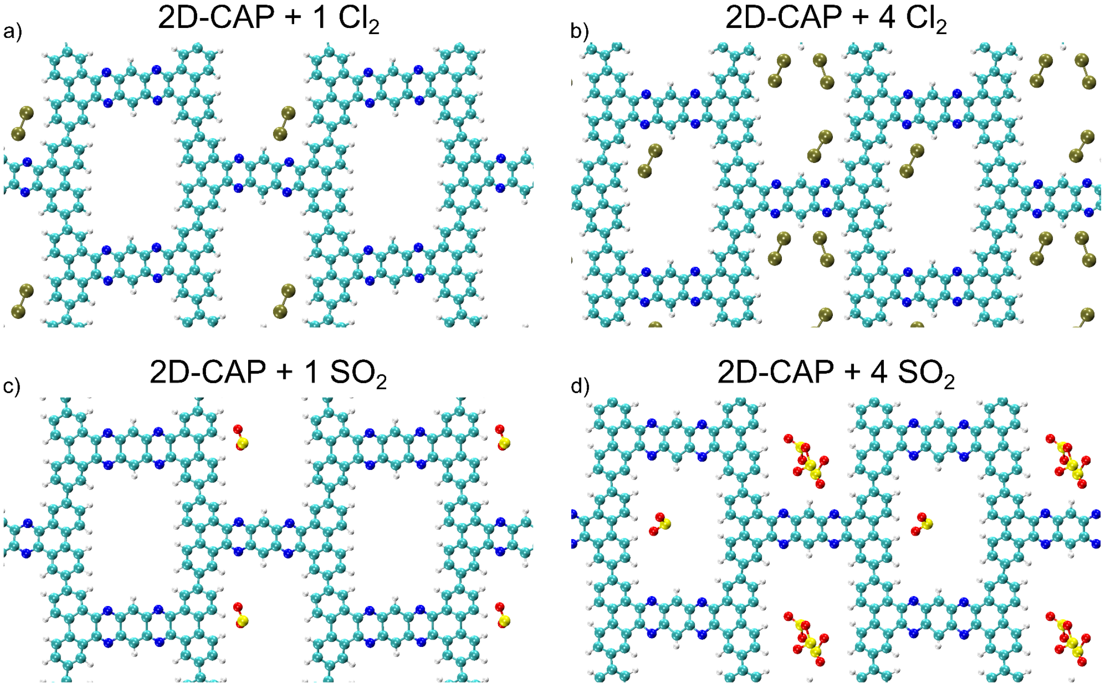

Figure S12. FARMD results for 2D-CAP with (a) Cl_2 and (b) SO_2. Carbon is cyan, nitrogen is blue, hydrogen is white, Oxygen is red, Sulfur is yellow and Chlorine is ochre.

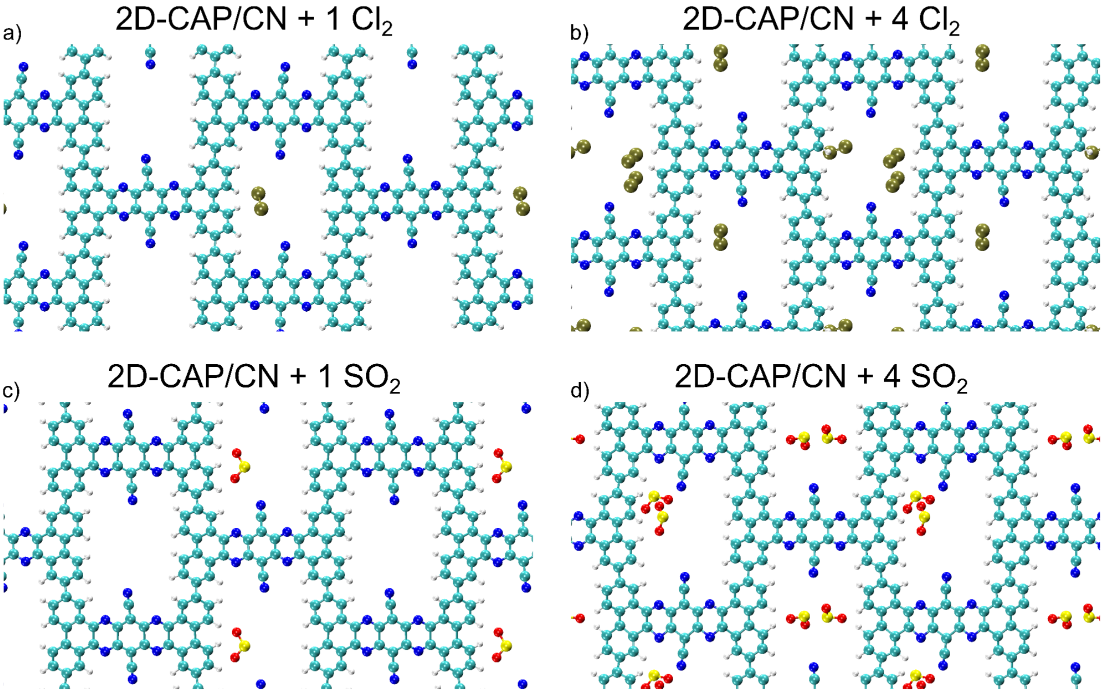

Figure S13. FARMD results for 2D-CAP/CN with (a-b) Cl_2 and (c-d) SO_2. Carbon is cyan, nitrogen is blue, hydrogen is white, Oxygen is red, Sulfur is yellow and Chlorine is ochre.

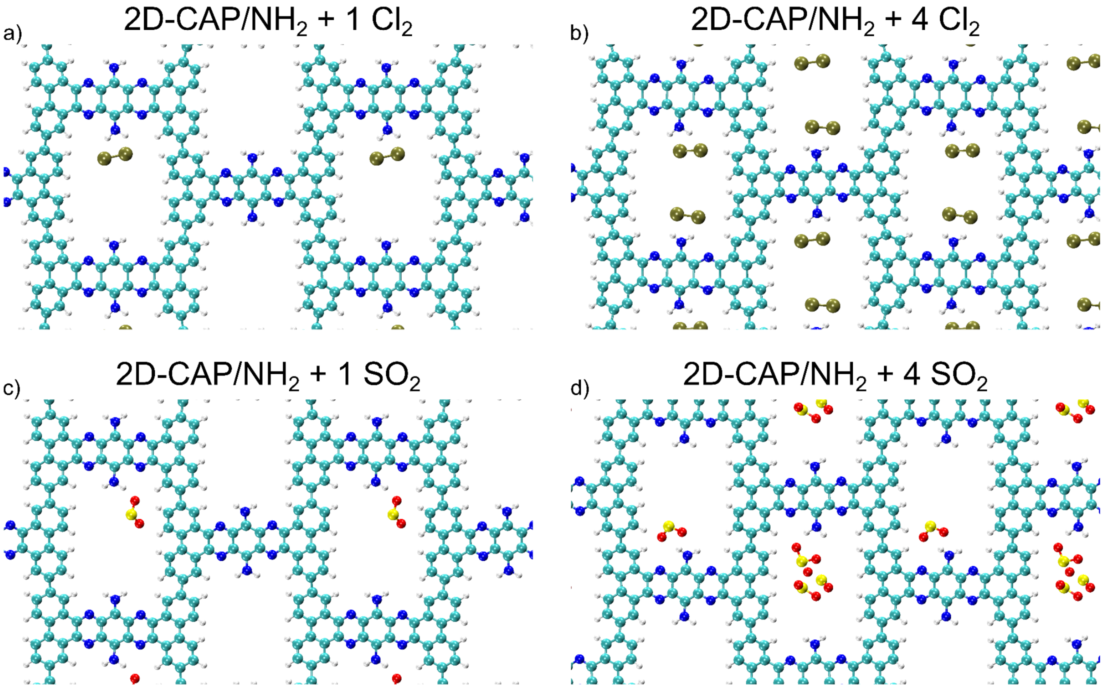

Figure S14. FARMD results for 2D-CAP/NH_2 with (a-b) Cl_2 and (c-d) SO_2. Carbon is cyan, nitrogen is blue, hydrogen is white, Oxygen is red, Sulfur is yellow and Chlorine is ochre.

Figures S15-S17 show the analyses of radial pair distributions considering distinct regions of 2D-CAP/R structures: horizontal segments (HS), vertical segments (VS) and side groups (SG) (as illustrated in Figure S12-S14), as well as projections on specific atoms of these regions (e.g. C_{HS} represent carbon atoms located at horizontal segments of 2D-CAP/R).

Hydrogen atoms play a key role in analyte adsorption. For 2D-CAP, Cl_2 adsorption is dominated by hydrogen atoms on the horizontal segments (especially H_{SG}), while SO_2 preferentially interacts with vertical segment hydrogens (H_{VS}). In 2D-CAP/CN, adsorption is mainly governed by H_{VS} for both analytes, with a secondary contribution from SG. For 2D-CAP/NH_2, adsorption is primarily driven by SG, with a weaker contribution from H_{VS}.

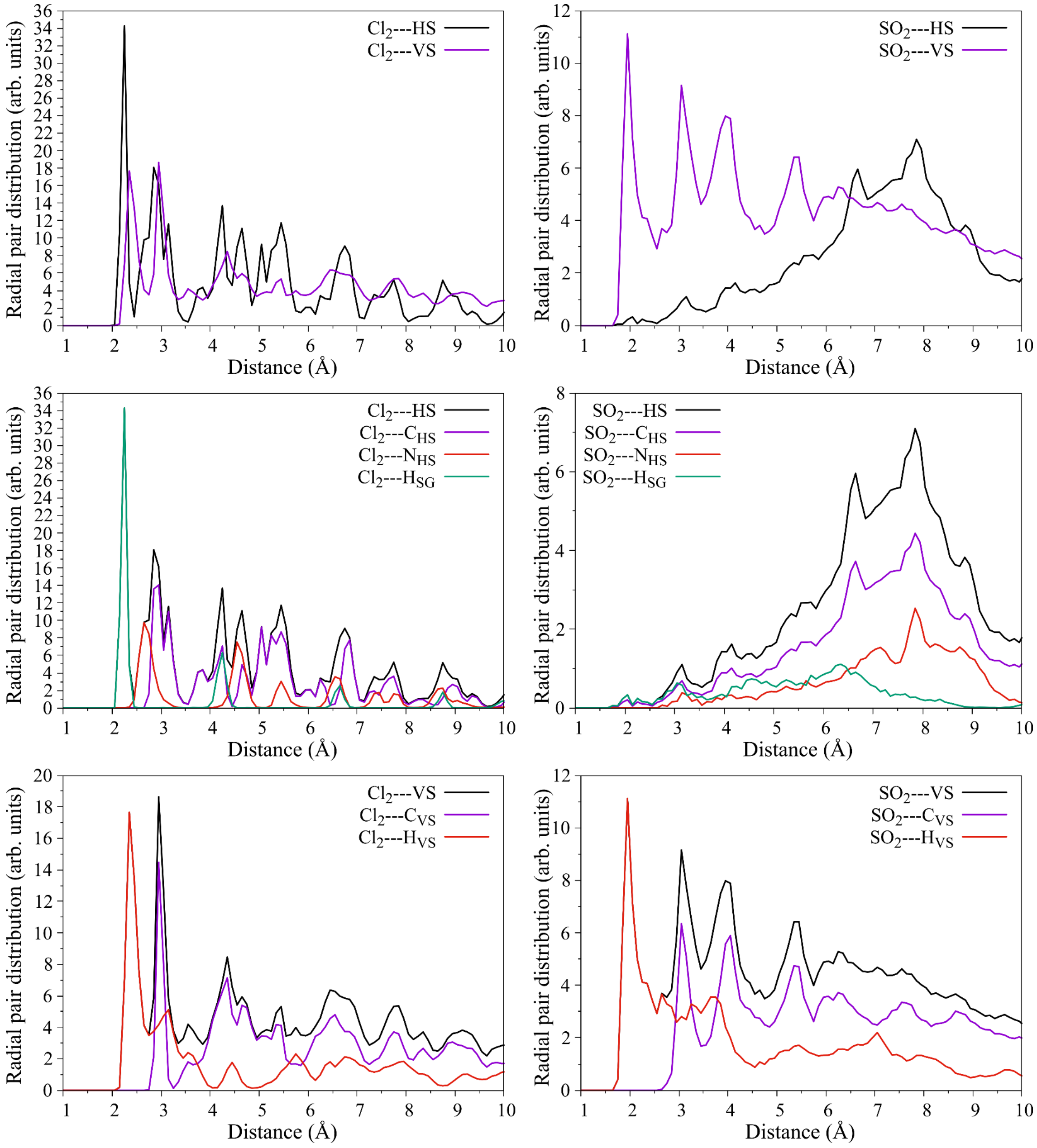

Figure S15. Radial distribution functions (RDFs) for 2D-CAP structures, resolved by structural regions: horizontal segments (HS), vertical segments (VS), and side groups (SG); and their corresponding atomic contributions.

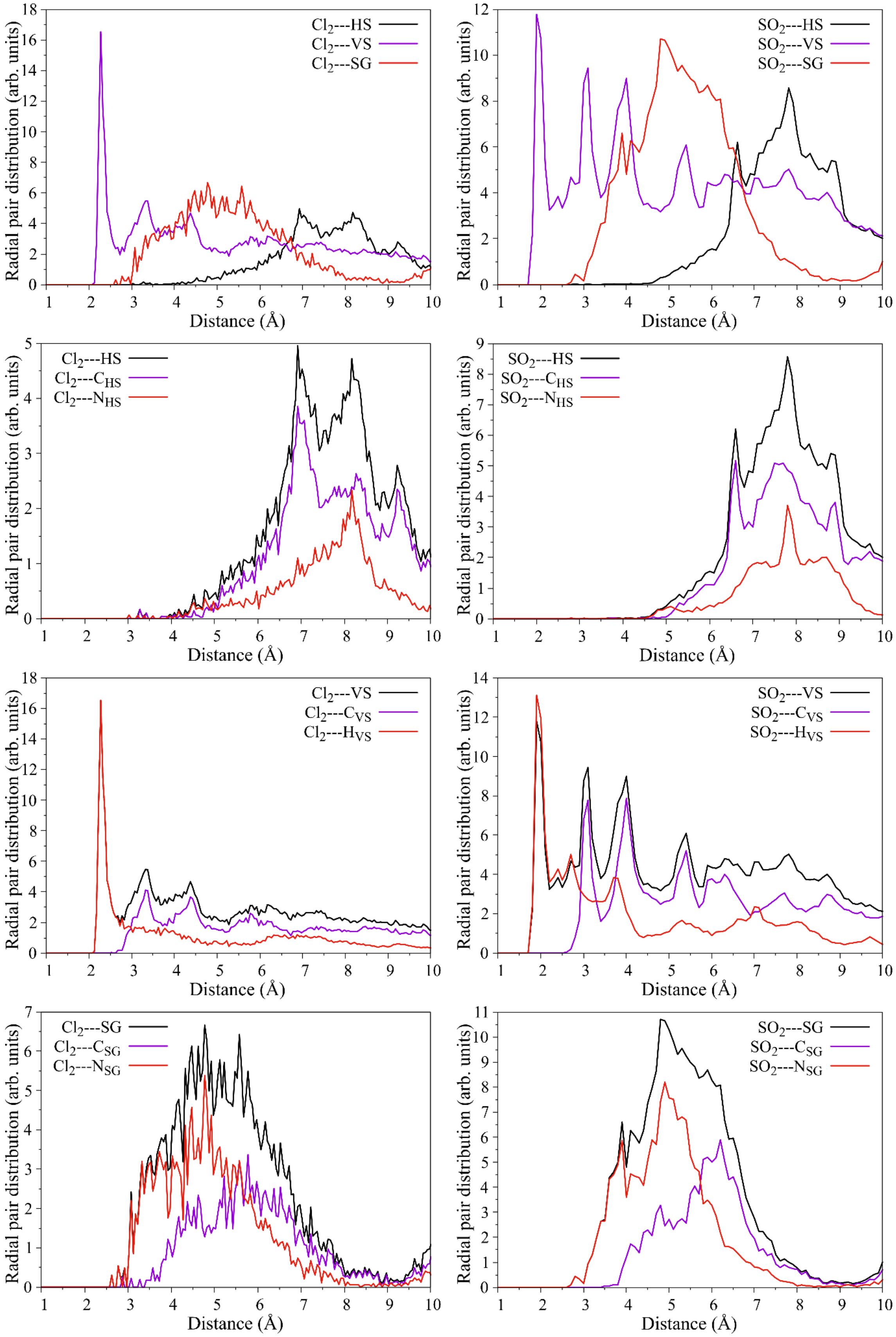

Figure S16. Radial distribution functions (RDFs) for 2D-CAP/CN structures, resolved by structural regions: horizontal segments (HS), vertical segments (VS), and side groups (SG); and their corresponding atomic contributions.

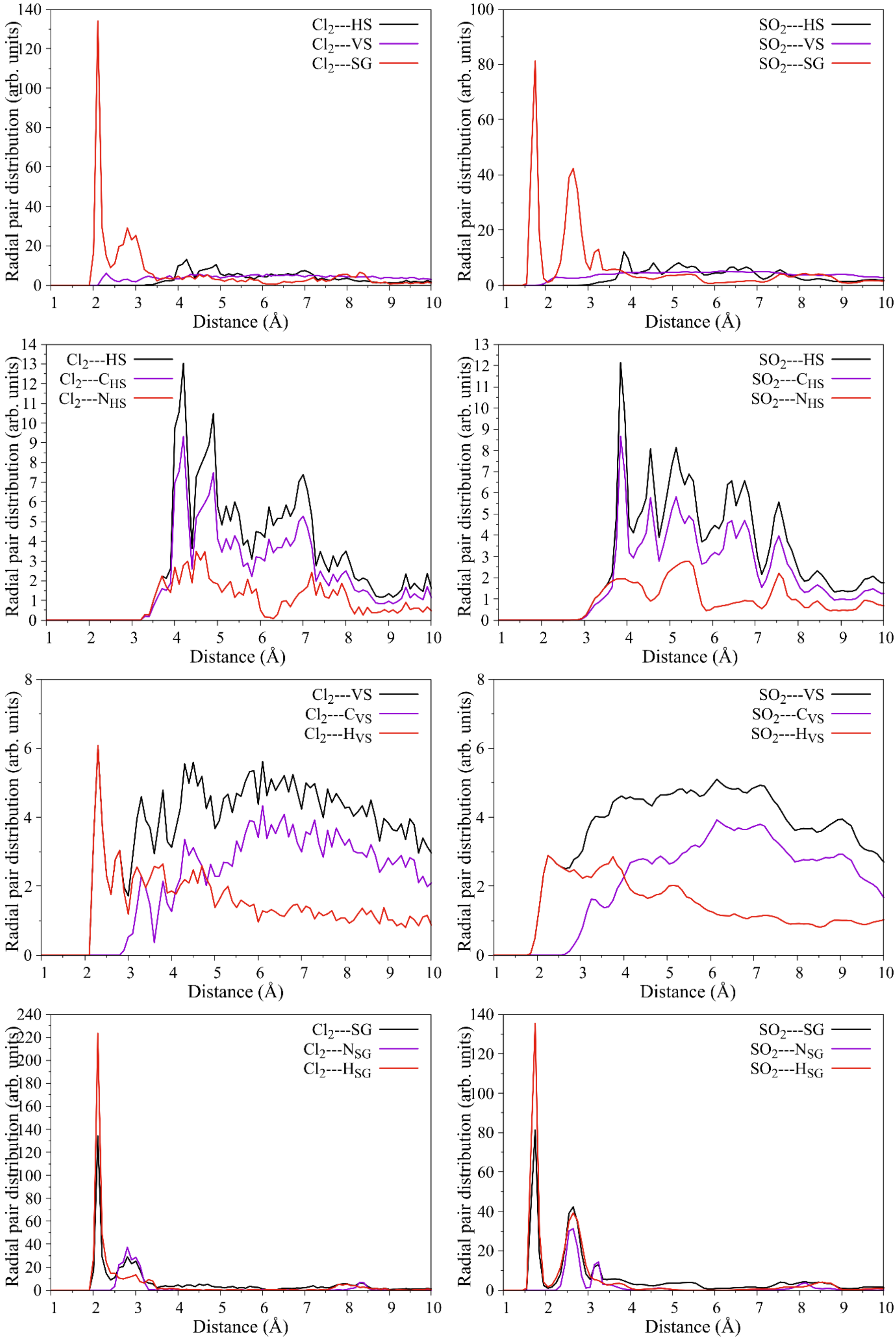

Figure S17. Radial distribution functions (RDFs) for 2D-CAP/NH_2 structures, resolved by structural regions: horizontal segments (HS), vertical segments (VS), and side groups (SG); and their corresponding atomic contributions.
